# Wireless Home Blood Pressure Monitoring System With Automatic Outcome-Based Feedback and Financial Incentives to Improve Blood Pressure in People With Hypertension: Protocol for a Randomized Controlled Trial

**DOI:** 10.2196/27496

**Published:** 2021-06-09

**Authors:** Marcel Bilger, Agnes Ying Leng Koong, Ian Kwong Yun Phoon, Ngiap Chuan Tan, Juliana Bahadin, Joann Bairavi, Ada Portia M Batcagan-Abueg, Eric A Finkelstein

**Affiliations:** 1 Health Economics and Policy Vienna University of Economics and Business Vienna, Austria Austria; 2 SingHealth Polyclinics Singapore Singapore; 3 Saudara Clinic by A+J General Physicians Singapore Singapore; 4 Heath Services and Systems Research Duke–NUS Medical School Singapore Singapore; 5 Duke Global Health Institute Duke University Durham, NC United States

**Keywords:** telemedicine, home blood pressure monitoring, behavior change, hypertension, financial incentive, medication adherence, remote titration

## Abstract

**Background:**

Hypertension is prevalent in Singapore and is a major risk factor for cardiovascular morbidity and mortality and increased health care costs. Strategies to lower blood pressure include lifestyle modifications and home blood pressure monitoring. Nonetheless, adherence to home blood pressure monitoring remains low. This protocol details an algorithm for remote management of primary care patients with hypertension.

**Objective:**

The objective of this study was to determine whether wireless home blood pressure monitoring with or without financial incentives is more effective at reducing systolic blood pressure than nonwireless home blood pressure monitoring (usual care).

**Methods:**

This study was designed as a randomized controlled open-label superiority study. A sample size of 224 was required to detect differences of 10 mmHg in average systolic blood pressure. Participants were to be randomized, in the ratio of 2:3:3, into 1 of 3 parallel study arms :(1) usual care, (2) wireless home blood pressure monitoring, and (3) wireless home blood pressure monitoring with financial incentives. The primary outcome was the mean change in systolic blood pressure at month 6. The secondary outcomes were the mean reduction in diastolic blood pressure, cost of financial incentives, time taken for the intervention, adherence to home blood pressure monitoring, effectiveness of the framing of financial incentives in decreasing nonadherence to blood pressure self-monitoring and the adherence to antihypertensive medication at month 6.

**Results:**

This study was approved by SingHealth Centralised Institutional Review Board and registered. Between January 24, 2018 and July 10, 2018, 42 participants (18.75% of the required sample size) were enrolled, and 33 participants completed the month 6 assessment by January 31, 2019.

**Conclusions:**

Due to unforeseen events, the study was stopped prematurely; therefore, no results are available. Depending on the blood pressure information received from the patients, the algorithm can trigger immediate blood pressure advice (eg, Accident and Emergency department visit advice for extremely high blood pressure), weekly feedback on blood pressure monitoring, medication titration, or skipping of routine follow-ups. The inclusion of financial incentives framed as health capital provides a novel idea on how to promote adherence to remote monitoring, and ultimately, improve chronic disease management.

**Trial Registration:**

ClinicalTrials.gov NCT 03368417; https://clinicaltrials.gov/ct2/show/NCT03368417

**International Registered Report Identifier (IRRID):**

DERR1-10.2196/27496

## Introduction

Hypertension is prevalent in Singapore, affecting 23.5% of adults between 18 to 69 years of age [1]. It is a major risk factor for cardiovascular morbidity and mortality [2,3] and is associated with significant health care cost [4,5]. The goal of hypertension management is to lower blood pressure to healthy ranges through lifestyle modifications such as restricting salt and alcohol intake, eating a healthy diet, losing weight, engaging in regular exercise, quitting smoking, and taking antihypertensive medication(s) [6].

For patients whose blood pressure remains high, doctors routinely recommend home blood pressure monitoring for better blood pressure control. Home blood pressure monitoring allows the doctor to monitor response to treatment, detect white-coat hypertension, and predict cardiovascular risk [7,8]. Nonetheless, adherence to home blood pressure monitoring and medication(s) remains low [9]. Even when patients adhere to home monitoring, the readings are not reviewed by the doctor until the patient’s subsequent in-person visit, which may be several weeks later. To address this, telemonitoring [10-14] can be employed as it allows for health care providers to monitor and intervene [15], to increase adherence to blood pressure monitoring [16], or titrate blood pressure medication [17] as needed, potentially without requiring an in-person visit [18]. However, systematic reviews [19,20] on telemonitoring reveal that it produces only modest improvements, which suggests that other features are needed [21-25]. For example, features such as automatic reminders [26,27], weekly feedback [28], or clinical interventions in response to concerning blood pressure trends (eg, medication titration), and financial incentives can be considered.

Behavioral economic theory suggests that the high rates of nonadherence to lifestyle modifications result, at least in part, from patients not perceiving a clear cause and effect relationship between greater adherence and reduced likelihood of adverse health consequences (eg, cardiovascular disease and premature death) [29,30]. The financial cost, such as the cost of medication(s), and nonfinancial costs, such as eating healthy, exercising, medication adherence, and blood pressure monitoring efforts, occur today, whereas the benefits, such as reduced risk for major cardiovascular events, often appear distant and uncertain. As a result, and because many individuals with hypertension feel perfectly healthy, they do not internalize the costs of nonadherence until it is too late. This theory suggests that a strategy to improve adherence is by providing a short-term financial incentive—an immediate benefit to offset the costs associated with the behavior change. Similar economic incentives have been used successfully in several adherence-enhancing interventions [31-34].

Therefore, this study aimed to leverage the potential of wireless and mobile technology and introduce financial incentives to improve the effectiveness of home blood pressure monitoring. The primary objective of this study was to determine whether wireless home blood pressure monitoring, with or without financial incentives, is more effective at reducing systolic blood pressure than nonwireless home blood pressure monitoring that relies on patient self-report (usual care). The secondary objectives were to improve adherence to blood pressure monitoring and antihypertensive medication(s).

## Methods

### Trial Design

The study was designed as a randomized controlled open-label superiority study with 3 parallel arms. Patients with hypertension who were on antihypertensive medication were randomized to (1) usual care, (2) wireless home blood pressure monitoring only, and (3) wireless home blood pressure monitoring with financial incentives arms, in a ratio of 2:3:3. Participants were randomly stratified based on whether they had diabetes mellitus (diabetes) and by clinic. The study intervention was to last 6 months. This protocol conforms to CONSORT (Consolidated Standards of Reporting Trials [35]) guidelines; the checklist can be found in Multimedia Appendix 1.

### Study Setting and Eligibility Criteria

Patients were recruited from Bedok and Marine Parade Polyclinics (SingHealth Polyclinics) in Singapore. Bedok and Marine Parade Polyclinics provide primary health care services to the eastern and southern region of Singapore. Hypertension management is one of the key services provided. Participant eligibility was based on the inclusion and exclusion criteria (Textbox 1).

**Inclusion and exclusion criteria.**

**Inclusion criteria**

Participants had to fulfil all of the following:

- Diagnosed hypertension and on at least 1 antihypertensive medication
- Systolic blood pressure ≥140 mmHg or diastolic blood pressure ≥90 mmHg for patients without diabetes (systolic blood pressure ≥140 mmHg or diastolic blood pressure ≥85 mmHg for patients with diabetes), which was verified by the average of the last 2 of 3 blood pressure readings taken on the day of the screening visit at 3-minute intervals [36] (model: HEM-7130, Omron)
- Age from 21 to 70 years of age
- Singapore citizens or permanent residents
- Able to converse in English
- Has a compatible smartphone (iOS: versions 8.0 and higher, Android: versions 5.0 and higher) with data plan or regular Wi-Fi access
- Ability to perform self-monitoring of blood pressure as assessed by the clinical research coordinator
- Expecting to be a patient of Bedok or Marine Parade Polyclinics for the duration of the trial

**Exclusion criteria**

Patients with any of the following were not enrolled:

- Systolic blood pressure ≥180 mmHg or diastolic blood pressure ≥110 mmHg which was verified by the average of the last 2 of 3 blood pressure readings taken on the day of the screening visit at 3-minute intervals (model: HEM-7130, Omron)
- Started on angiotensin-converting enzyme inhibitors or angiotensin-receptor blockers within the last 3 months
- Clinically unstable heart failure
- Advanced kidney disease (estimated glomerular filtration rate <30 mL/minute CKD-EPI Creatinine formula [37]
- Acute kidney injury (ie, increase in serum creatinine ≥50% from baseline within the past week
- Confirmed glomerulonephritis
- Severe or overt macro albuminuria (urine albumin-to-creatinine ratio >30 mg/mmol or protein-to-creatinine ratio >0.5)
- Known liver disease (eg, liver cirrhosis)
- Atrial fibrillation
- On warfarin or anticoagulants (eg, novel oral anticoagulants)
- Underwent double mastectomy
- Pregnant
- Known allergy to epoxy resin
- Newly referred to specialist outpatient clinics or upon follow-up for complications related to hypertension
- Discharged from hospital within the last 3 months for complications related to hypertension
- Any other major debilitating disease or mental illness that precludes validity of informed consent or would result in the patient being unable to take their blood pressure independently
- Living in a household where another member has been recruited into the trial

### Participant Recruitment, Timeline, and Study Arms

#### Overview

Participants were recruited via posters and referrals at Bedok and Marine Parade Polyclinics. A screening visit was arranged during which the study’s purpose was explained and the screener administered. For eligible patients, the clinical research coordinator went through the participant information sheet, and if the patient agreed to participate, obtained informed consent (Multimedia Appendix 2).

Blood pressure was assessed at baseline and at month 6. Participants wore an ambulatory blood pressure monitor (model 7100, Welch Allyn) for 12 hours while awake (eg, 9 AM-9 PM). A period of 12 hours instead of 24 hours was chosen to reduce participant burden [38]. A diary was also given to participants to record antihypertensive medication adherence and physical activity (Multimedia Appendix 3, Figure S1). Participants were also issued a home blood pressure monitor and advised to monitor their blood pressure 3 times per week during the study. Medication Tracker (eCAP) was also issued, and participants were advised to store their most frequently prescribed antihypertensive medication in it. A demonstration of the study devices was given. The baseline questionnaire was administered (in paper format) at Bedok and Marine Parade Polyclinics, and the participant’s responsibilities and adherence goals explained. The study intervention began on the Monday following the valid baseline ambulatory blood pressure monitoring test (defined as having at least 70% of successful readings [39]). Participants also completed questionnaires at the 6-month assessment. Multimedia Appendix 4 contains the study timeline.

#### Arm 1: Usual Care

SingHealth Polyclinics have a structured framework for hypertension management. Patients who are newly diagnosed with hypertension would be prescribed antihypertensive medication if deemed necessary by the doctor. All patients are subsequently referred to a nurse who would provide further information on hypertension and come up with a lifestyle modification plan with the patient. Patients are then followed up by the doctors and nurses at 3- to 4-month intervals (or more), based on their blood pressure trend. Further education is given at these visits as needed. Patients with good blood pressure control can teleconsult with a trained nurse, alternating with in-person doctor's consultation at up to 6-month intervals if they monitor their blood pressure at home. There are in-house pharmacists who assist patients in understanding their medication doses and regimens. Regular blood tests (ie, electrolytes, renal function, lipids, and glucose) are also carried out annually to monitor the patient’s response to treatment and to detect any disease progression and complications. Patients with evidence of disease progression and complications would be closely monitored and referred to the appropriate specialists if required.

Participants in the usual care arm were advised to use their existing blood pressure monitor. Participants who did not have a home blood pressure monitor were given a blood pressure monitor (HEM 7130, Omron). In order to properly identify the effect of contingent financial incentives, all participants received a participant leaflet (Textbox 2).

The clinical research coordinator also provided advice on self-management and education on how to interpret blood pressure readings according to a standard self-monitoring guideline (Multimedia Appendix 5). The self-monitoring instructions were adapted from the guidelines of the Healthy Singapore website, a website by the Singapore Ministry of Health which was discontinued in September 2016. Elevated blood pressure was defined as clinic-measured systolic blood pressure 140 mmHg or diastolic blood pressure 90 mmHg. Published home-based monitoring protocols however, state that for home blood pressure monitoring, systolic blood pressure ≥135 mmHg or diastolic blood pressure ≥85 mmHg is indicative of elevated blood pressure [40,41]. In addition, SingHealth Polyclinics guidelines recommend home blood pressure monitoring targets of <135/80 mmHg for patients with diabetes, and <135/85 mmHg for those without diabetes. We therefore worked with the SingHealth Polyclinics Telehealth Team to fine-tune the blood pressure cut-offs for the various categories in order to have the same blood pressure cut-offs as the intervention (Table 1).

Participants in Arm 1 recorded their blood pressure readings on the SingHealth Polyclinics home blood pressure charting form (in paper format) as part of usual care. For monitoring adherence to hypertensive medicines, an eCAP (Information Mediary Corp) was used. The eCAP device passively recorded the dates and times the bottle was opened; these data are stored in the memory via an radiofrequency identification tag. Data were extracted by scanning the eCAP device on a reader (CertiScan desktop). Participants were assessed for adherence within specified time windows (eg, if a participant’s specified timing is 5 AM to 11 AM and 5 PM to 11 PM, a reading had to be logged within both windows for the participant to be considered adherent for that day).

**Participant leaflet.**

Aim to achieve blood pressure readings in the normal range

Measure your blood pressure on at least 3 days each week

Recommendations:

1. It is best to measure your blood pressure in the morning, before you take your medication, coffee, tea, smoke or exercise. Please sit for at least 5 minutes before measuring your blood pressure.
2. It is also recommended that you measure your blood pressure before you sleep at night.

Get active

Eat better. Reduce your salt intake

Take your medication as prescribed by your doctor.

**Table 1:** Blood pressure classification.

Diabetes status			Very low		Low normal		Normal		Slightly high		Very high		Extremely high
**No diabetes**													
	Systolic blood pressure (mmHg)	<90		90-99		100-134		135-159		160-179		≥180	
	Diastolic blood pressure (mmHg)	<50		50-59		60-84		85-99		100-109		≥110	
**Diabetes**													
	Systolic blood pressure (mmHg)	<90		90-99		100-134		135-159		160-179		≥180	
	Diastolic blood pressure (mmHg)	<50		50-59		60-79		80-99		100-109		≥110	

#### Arm 2: Wireless Home Blood Pressure Monitoring System

**Figure 1:**
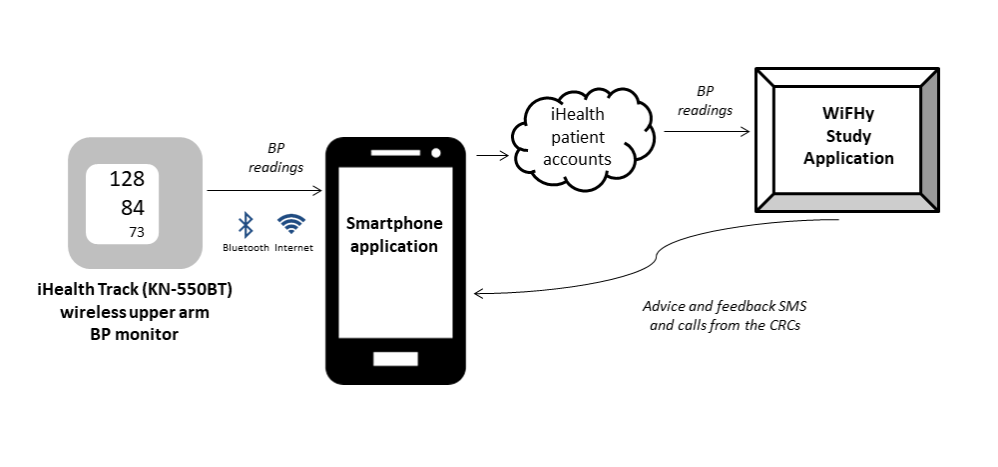
Wireless home blood pressure monitoring system. BP: blood pressure; CRC: Clinical Research Coordinator; WiFHy: Wireless Monitoring and Financial Incentives for Uncontrolled Hypertension.

Participants in Arm 2 used an asynchronous telehealth system that consisted of a wireless home blood pressure monitor and app (Figure 1). The study app comprised 3 parts: (1) instant home blood pressure monitoring advice, (2) weekly home blood pressure monitoring adherence feedback, and (3) 28-day continuous home blood pressure monitoring assessment.

The participants monitored their blood pressure using a wireless upper arm blood pressure monitor (iHealth KN-550BT [42]). The blood pressure monitor transmitted the readings via Bluetooth and internet to a smartphone app on the participants’ smartphones. Participants received training from the clinical research coordinator on how to use the device and to upload their blood pressure readings. This smartphone app was available at no charge to study participants. Data were automatically sent to secure participant accounts, then pushed to a secure study app. The study app then sent feedback SMS text messages to the participants and automatically triggered interventions from the polyclinics depending on the blood pressure readings (Table 2).

**Table 2:** Blood pressure–related procedures.

Part	Description
Part 1: Instant home blood pressure monitoring advice (Multimedia Appendix 6)	Each blood pressure reading was classified in the order of most abnormal to normal (ie extremely high, very high, slightly high, very low, low normal and normal) and displayed on the study website. Based on the blood pressure classification, the participant received SMS self-management advice. A colour coded protocol (Red protocol)^a^ was activated for very low and extremely high blood pressure readings.
Part 2: Weekly home blood pressure monitoring adherence feedback (Multimedia Appendix 7)	Participants received automated praise, encouragement, or reminder SMS messages on the Monday of the following week at 8 AM throughout the intervention, with content that were dependent on their adherence to home blood pressure monitoring the week prior.
Part 3: 28-day continuous home blood pressure monitoring assessment (Multimedia Appendix 8)	The average of blood pressure readings over the past 28 days was calculated daily based on readings over the preceding 28 days. It was categorized in the order of most abnormal to normal and color coded^a^. The triggering of interventions based on the average blood pressure in the past 28 days is in line with previous studies that recommend using the average of a series of measurements for clinical decisions [43]. The 28-day continuous home blood pressure monitoring assessment occurred when the system detected a minimum of 8 blood pressure readings in the past 28 days.

^a^Color-coded protocols (Multimedia Appendix 8)—red: for very low and extremely high blood pressure; black: for very low or extremely high average blood pressure; gray: for low normal average blood pressure; green: for average blood pressure within the normal range just before scheduled clinic visit (participants were eligible to skip their upcoming clinic visit on approval of a doctor after review of the participant’s clinical history and verification of the participant’s current well-being by the clinical research coordinator); pink: for slightly high or very high average blood pressure (remote drug titration for participants on selected drugs); yellow: no readings for the past 28 days (the clinical research coordinator contacted the participant to determine the reason).

#### Remote Titration (Arm 2 and 3 Participants Only)

To be clinically more responsive to uncontrolled blood pressure, remote titration is integrated in this intervention as per the Joint National Committee 8 [44] recommendation to increase the dose of an initial drug if goal blood pressure is not reached within 1 month of treatment. This protocol for drug titration is based on an unpublished pilot program at Pasir Ris Polyclinic. Participants were eligible for remote titration if they were randomized to Arms 2 and 3 and prescribed (1) nifedipine LA ≤30 mg/day, (2) amlodipine ≤7.5 mg/day, (3) atenolol ≤75 mg every morning or (4) bisoprolol ≤7.5 mg every morning at baseline. For participants who were on more than 1 drug, the study doctor determined the drug to be titrated based on the drug selection workflow (Multimedia Appendix 8, Figure S4) and gave instructions on the dose increase to the participant. The participant was to increase the dose only if contacted by the clinical research coordinator to do so during the intervention. The doctor’s instructions were also written in an individualized leaflet (Remote Titration Action Plan–Patient Information Leaflet; Multimedia Appendix 8, Figure A8.4.1.2) and given to the participant for reference. The clinical research coordinator also tagged the drug to be titrated and reinforced the doctor’s advice. Participants were prescribed the full duration of the antihypertensive drugs until their next scheduled clinic visit. The drugs selected for remote titration were calcium channel blockers and beta blockers as these do not require monitoring of electrolytes.

On each working day, the clinical research coordinator logged-in to the study website, monitored the dashboard for flags and intervened accordingly. The clinical research coordinator unflagged the flag once the intervention had been carried out.

Study participants had doctor consultations at month 3 and at month 6 during the intervention period. For Arm 1 participants, the clinical research coordinator met with them prior to their doctor consultation and made a copy of their blood pressure readings for record. For Arm 2 and 3 participants, the clinical research coordinator passed them the blood pressure readings captured by the wireless monitoring system on the day of the doctor consultation for review by the doctor; except in cases where the visit was skipped due to good blood pressure control (green protocol).

#### Arm 3: Wireless Home Blood Pressure Monitoring System With Incentives

Participants in this arm received an intervention identical to those in Arm 2, with the addition of financial incentives for blood pressure monitoring. This arm was subdivided into 2 arms; participants were eligible to receive the same incentive amounts but framed differently. In the instant reward subarm, participants received SGD $3 (an exchange rate of approximately SGD $1 to US $0.75 was applicable at the time of publication) for each day they measured their blood pressure, up to 3 times per week (SGD $9 if they measured their blood pressure on at least 3 different days, SGD $6 if they measured their blood pressure on at least 2 different days, SGD $3 if they measured their blood pressure on at least 1 day, or no financial incentive if they did not measure their blood pressure). In the health capital subarm, participants received an initial health capital of SGD $72. Participants’ health capital increased by SGD $6 on each week they measured their blood pressure on at least 3 different days. Participants’ health capital decreased weekly by 10% per missing blood pressure reading. Health capital decreased by 10% if the participant measured their blood pressure on 2 different days, by 20% if the participant measures their blood pressure only on 1 day, and by 30% if the participant did not measure their blood pressure. Therefore, at the end of the 24-week intervention, Arm 3 participants could receive incentives up to SGD $216 for blood pressure monitoring.

Noncontingent study payments (Multimedia Appendix 9) to Arm 1 and 2 participants and incentive payments to Arm 3 participants (Multimedia Appendix 7, Table A7.4) in the form of supermarket vouchers were disbursed by the clinical research coordinator at the month 6 assessment.

### Participant Withdrawal

During the study intervention, the clinical research coordinator reviewed the participants’ medical records and contacted the participants via phone to determine if there were any serious adverse events, changes in medical condition, hospitalizations, or referrals to specialist outpatient clinics that may require a withdrawal from the study. The study doctor reviewed the participant’s medical records and determined when there was a need to do so. Participants who were newly diagnosed with a hypertension related condition or complication or met the following exclusion criteria: clinically unstable heart failure, acute kidney injury, glomerulonephritis, liver disease, atrial fibrillation, prescribed warfarin or anti-coagulants, double mastectomy, pregnant, epoxy-resin allergy, referred to specialist outpatient clinic, or hospitalized for complications were withdrawn. Those who had a progression of an existing condition (eg, impaired kidney function) could remain in the study. For study participants who were withdrawn by the study team, a payment of SGD $80 in compensation for forgoing potential payments that the participant might have received had they remained in the study was given. Participants were free to withdraw their consent and discontinue their participation at any time during the intervention, without prejudice or effect on their medical care; however, all data collected until the time of the participants’ withdrawal were kept to allow for a comprehensive evaluation.

### Outcome Measures

The outcome measures and schedule of collection can be found in Multimedia Appendix 10.

#### Primary Outcome

The primary outcome was the mean reduction in systolic blood pressure in 6 months. This was to be obtained from the participants’ month 6 ambulatory blood pressure monitor results. systolic blood pressure is associated with increased risk of cardiovascular disease [45-47].

#### Secondary Outcomes

Secondary outcomes were mean reduction in diastolic blood pressure in 6 months, obtained from the participants’ month 6 ambulatory blood pressure monitor results; mean cost of financial incentives at month 6, calculated as the total financial incentives earned by Arm 3 participants at the end of the intervention and to be used as part of the cost-effectiveness analysis; mean time taken for the intervention at month 6, calculated as the total number of minutes spent by the clinical research coordinator intervening for the colored flags, adherence calculation, and payment of financial incentives for all study arm participants during the month 6 assessment and to be used as part of the cost-effectiveness analysis; mean adherence to home blood pressure monitoring at month 6; effectiveness of the framing of financial incentives in decreasing nonadherence to blood pressure self-monitoring; and mean adherence to antihypertensive medication (prescribed to be taken most frequently) at month 6.

Diastolic blood pressure may have some associations with future systolic hypertension and with increased risk of cardiovascular disease [47-51].

#### Exploratory Outcomes

Exploratory outcomes were the proportion of participants who have target blood pressure (defined as less than 130 mmHg/80 mmHg) at month 6; mean change from baseline in European Quality of Life-5 Dimensions-5 Levels [52] score at month 6; mean change from baseline in Brief Illness Perception Questionnaire [53] score, which assesses perceptions of hypertension, at month 6; mean change from baseline in the Global Physical Activity Questionnaire at month 6; mean change from baseline in the Dietary Practices Questionnaires [54] at month 6; mean change from baseline in Healthcare Services Expenditure at month 6; and the Treatment Satisfaction on home blood pressure monitoring, which is a modified version of the Treatment Satisfaction Questionnaire for Medication [55], at month 6.

### Sample Size

A key parameter is the systolic blood pressure. To assess this, we computed the sample size to be able to detect differences of 10 mmHg in average systolic blood pressure between study arms at the 5% level with 80% power. To compute the overall sample size, we computed the size of the intervention groups that is required for testing for difference between intervention groups (study arms 2 and 3). After applying a Bonferroni correction (by dividing the test’s significance level by 2, which is the number of comparisons that we test in our study for the primary outcome), we found that 68 patients per intervention group is necessary to detect mean differences in systolic blood pressure of 10 mmHg (with 2.5% significance level and 80% power). Given the resulting cumulated sample size for the 2 intervention arms of 136, we then computed the size required for the control group to test the overall effect of the wireless home blood pressure monitoring (with and without financial incentives) with the same effect size, significance level, and power. This computation yielded a size of 45 for the control group. After accounting for 25% attrition in each arm, the resulting sample sizes were 56 for the control group and 84 for each intervention group, resulting in a total of 224 patients. We assumed throughout that the standard deviation of systolic blood pressure is 19 (which is slightly greater than the maximum value reported by a similar size 6-month study of patients with uncontrolled hypertension [56]).

### Randomization

Participants were allocated to 1 of 3 study arms by random assignment. Prior to recruitment, randomization numbers was generated by the principal investigator using Stata software (StataCorp LLC) to create an assignment schedule for block-randomization to allocate eligible participants into 1 of the 3 study arms in a ratio of 2:3:3. Randomized stratification was based on whether the patient had diabetes and study site. The block size was not communicated to SingHealth Polyclinics to minimize the predictability of the random sequence. Furthermore, in order to test a secondary hypothesis (H5), the patients in the home blood pressure monitoring with financial incentives arm were further randomly divided into 2 equal-size groups, 1 per incentive type. The project coordinator and principal investigator then stored the assignment schedule on a secure server at Duke–NUS Medical School. For allocation concealment, the project coordinator and a Duke–NUS Medical School staff external to the study team enclosed the assignments in sequentially numbered, opaque, sealed randomization envelopes. These were handed over to the clinical research coordinator for participant enrollment and assignment.

### Allocation Blinding

The clinical research coordinators were not blinded to the group allocation during the study intervention as there was a need for the clinical research coordinators to know the participants’ arms to assign study devices to the participants and carry out the intervention accordingly and disburse payouts for the incentive arm participants (Arm 3). The site investigators were also not blinded as they had to explain the remote titration action plan and advise on intervention for the colored protocols (Arm 2 and 3).

### Data Management and Monitoring

To maintain confidentiality, all enrolled participants were issued a unique ID based on their randomization number and were referred to via their unique ID thereon. Identifiable data were kept at locked cabinets at Bedok and Marine Parade Polyclinics and only accessible by the SingHealth Polyclinics team. Only deidentified research data were passed to Duke–NUS Medical School, and all data transfers were documented. All keyed in deidentified research data were encrypted, password-protected and stored on a secure server. Blood pressure readings and home blood pressure monitoring adherence data from the mobile app were transmitted automatically to the app via the application programming interface daily. The app did not contain any identifying information, was password protected, and only authorized members of the Duke–NUS Medical School team had access to the website via 2-factor authentication. Investigators have access to the research data collected. All hardcopy research data collected are archived for the next 10 years in compliance with NUS's research data management policies. No Data and Safety Monitoring Board was used for this trial as the study was deemed to be low risk by the investigators as this intervention was modelled after an existing standard of care by the Polyclinics and not involving more than minimal risk to the participants. Compensation was to be considered on a case-by-case basis for unexpected injuries due to nonnegligent causes. This trial was subjected to study review visits and audits to ensure that all investigator-initiated research is conducted effectively and efficiently.

### Ethics

This study was approved by the SingHealth Centralised Institutional Review Board E (2016/2026). Amendments to the protocol or other study-related documents were approved by the institutional review board.

### Data Analysis

All main analyses were to be based on intent to treat. The mean difference in the systolic blood pressure at 6 months was to be assessed in the context of a linear mixed-effects model with a random effect for subject and fixed effects for baseline mean (the same in both arms) and change in mean at 6 months for each treatment arm (one for each arm). The mixed-effects model allows nonmissing data to be used in analysis without imputation. For estimates of the treatment arm effects to be unbiased, data must be missing-at-random. However, it is possible that the analysis of the pattern of attrition would indicate that additional covariates needed to be included in the mixed-effects models. If data are not normally distributed, appropriate transformations will be attempted before resorting to using nonparametric statistical analysis methods.

## Results

Recruitment at Bedok Polyclinic began on January 24, 2018 and at Marine Parade Polyclinic on June 26, 2018. From January 24, 2018 to July 10, 2018, 42 participants (18.75% of the total sample size) were enrolled, and 33 participants completed the 6-month assessment by January 31, 2019. A decision was made to terminate the study prematurely due to unforeseen delays and resulting funding issues. No analysis was carried out due to the lack of sample size and therefore no results are available.

## Discussion

This paper reports a protocol for a randomized trial to determine whether a wireless home blood pressure monitoring system, with or without financial incentives, is more effective at reducing blood pressure than a nonwireless home blood pressure monitoring that relies on patient self-report or best practices. 

While the 6-month study intervention is sufficient to detect potential blood pressure improvement that is clinically significant, a longer period would be needed to test for the long-term effectiveness of the intervention. There is currently no prior intervention that has the same components as those in our wireless home blood pressure monitoring system.

Unfortunately, due to unforeseen events the study was stopped prematurely. Regardless, as this study raises an interesting set of research questions, this protocol may be of value to other researchers considering similar efforts for blood pressure control or other behavioral targets. Results of such a study would provide evidence on whether a telemonitoring system with or without financial incentives can improve hypertension management, thereby reducing long-term complications and health care cost. By interacting socioeconomic characteristics with the intervention effect, this study may also have provided evidence on the benefit incidence of interventions involving financial incentives. The framing of financial incentives as reward versus health capital, would also have informed the design of future incentive strategies in hypertension management and other chronic diseases. The study would also have added to telemonitoring knowledge on hypertension self-management in patients and remote clinical management of hypertension, which is increasingly relevant in light of COVID-19 and an increase in the use of telehealth in managing chronic diseases.

## Appendix

Multimedia Appendix 1 CONSORT E-HEALTH checklist.

Multimedia Appendix 2 Participant Information Sheet and Consent Form.

Multimedia Appendix 3 Ambulatory Blood Pressure Monitoring.

Multimedia Appendix 4 Study timeline for participants.

Multimedia Appendix 5 Standard guideline for interpretation of blood pressure readings.

Multimedia Appendix 6 Instant home blood pressure monitoring advice.

Multimedia Appendix 7 Weekly home blood pressure adherence feedback.

Multimedia Appendix 8 The 28-day continuous home blood pressure monitoring assessment.

Multimedia Appendix 9 Study payment scheme.

Multimedia Appendix 10 Outcome measures and schedule of collection.

Multimedia Appendix 11 Peer-review report by the National Medical Research Council (Ministry of Health, Singapore).

## Abbreviations

SGD: Singapore dollar
SMS: short message service

## References

[1] (2011). National Health Survey 2010. Ministry of Health Singapore.

[2] MacMahon S, Peto R, Cutler J, Collins R, Sorlie P, Neaton J, Abbott R, Godwin J, Dyer A, Stamler J (1990). Blood pressure, stroke, and coronary heart disease. part 1, prolonged differences in blood pressure: prospective observational studies corrected for the regression dilution bias. Lancet.

[3] Collins R, Peto R, MacMahon S, Hebert P, Fiebach NH, Eberlein KA, Godwin J, Qizilbash N, Taylor JO, Hennekens CH (1990). Blood pressure, stroke, and coronary heart disease. part 2, short-term reductions in blood pressure: overview of randomised drug trials in their epidemiological context. Lancet.

[4] Benjamin EJ, Blaha MJ, Chiuve SE, Cushman M, Das SR, Deo R, de FSD, Floyd J, Fornage M, Gillespie C, Isasi CR, Jiménez MC, Jordan LC, Judd SE, Lackland D, Lichtman JH, Lisabeth L, Liu S, Longenecker CT, Mackey RH, Matsushita K, Mozaffarian D, Mussolino ME, Nasir K, Neumar RW, Palaniappan L, Pandey DK, Thiagarajan RR, Reeves MJ, Ritchey M, Rodriguez CJ, Roth GA, Rosamond WD, Sasson C, Towfighi A, Tsao CW, Turner MB, Virani SS, Voeks JH, Willey JZ, Wilkins JT, Wu JH, Alger HM, Wong SS, Muntner P, American HASCSS (2017). Heart disease and stroke statistics-2017 update: a report from the American Heart Association. Circulation.

[5] Wang G, Grosse SD, Schooley MW (2017). Conducting research on the economics of hypertension to improve cardiovascular health. Am J Prev Med.

[6] (2017). Clinical practice guidelines: hypertension. Ministry of Health Singapore.

[7] George J, MacDonald T (2015). Home blood pressure monitoring. Eur Cardiol.

[8] Armstrong C, Joint National Committee (2014). JNC8 guidelines for the management of hypertension in adults. Am Fam Physician.

[9] Tan NC, Khin LW, Pagi R (2005). Home blood-pressure monitoring among hypertensive patients in an Asian population. J Hum Hypertens.

[10] Green BB, Cook AJ, Ralston JD, Fishman PA, Catz SL, Carlson J, Carrell D, Tyll L, Larson EB, Thompson RS (2008). Effectiveness of home blood pressure monitoring, web communication, and pharmacist care on hypertension control: a randomized controlled trial. JAMA.

[11] Bosworth HB, Powers BJ, Olsen MK, McCant F, Grubber J, Smith V, Gentry PW, Rose C, Van Houtven C, Wang V, Goldstein MK, Oddone EZ (2011). Home blood pressure management and improved blood pressure control: results from a randomized controlled trial. Arch Intern Med.

[12] McManus RJ, Mant J, Bray EP, Holder R, Jones MI, Greenfield S, Kaambwa B, Banting M, Bryan S, Little P, Williams B, Hobbs FDR (2010). Telemonitoring and self-management in the control of hypertension (TASMINH2): a randomised controlled trial. Lancet.

[13] Rinfret S, Lussier M, Peirce A, Duhamel F, Cossette S, Lalonde L, Tremblay C, Guertin M, LeLorier J, Turgeon J, Hamet P (2009). The impact of a multidisciplinary information technology-supported program on blood pressure control in primary care. Circ Cardiovasc Qual Outcomes.

[14] Franssen M, Farmer A, Grant S, Greenfield S, Heneghan C, Hobbs R, Hodgkinson J, Jowett S, Mant J, Martin U, Milner S, Monahan M, Ogburn E, Perera-Salazar R, Schwartz C, Yu L, McManus RJ (2017). Telemonitoring and/or self-monitoring of blood pressure in hypertension (TASMINH4): protocol for a randomised controlled trial. BMC Cardiovasc Disord.

[15] Omboni S, Ferrari R (2015). The role of telemedicine in hypertension management: focus on blood pressure telemonitoring. Curr Hypertens Rep.

[16] AbuDagga A, Resnick HE, Alwan M (2010). Impact of blood pressure telemonitoring on hypertension outcomes: a literature review. Telemed J E Health.

[17] Chen T, Kao C, Cheng S, Chang Y (2019). Effect of home medication titration on blood pressure control in patients with hypertension: a meta-analysis of randomized controlled trials. Med Care.

[18] Currell R, Urquhart C, Wainwright P, Lewis R (2000). Telemedicine versus face to face patient care: effects on professional practice and health care outcomes. Cochrane Database Syst Rev.

[19] Bray EP, Holder R, Mant J, McManus RJ (2010). Does self-monitoring reduce blood pressure? meta-analysis with meta-regression of randomized controlled trials. Ann Med.

[20] Fahey T, Schroeder K, Ebrahim S (2005). Interventions used to improve control of blood pressure in patients with hypertension. Cochrane Database Syst Rev.

[21] Bosworth HB, Olsen MK, Grubber JM, Neary AM, Orr MM, Powers BJ, Adams MB, Svetkey LP, Reed SD, Li Y, Dolor RJ, Oddone EZ (2009). Two self-management interventions to improve hypertension control: a randomized trial. Ann Intern Med.

[22] Tucker KL, Sheppard JP, Stevens R, Bosworth HB, Bove A, Bray EP, Earle K, George J, Godwin M, Green BB, Hebert P, Hobbs FDR, Kantola I, Kerry SM, Leiva A, Magid DJ, Mant J, Margolis KL, McKinstry B, McLaughlin MA, Omboni S, Ogedegbe O, Parati G, Qamar N, Tabaei BP, Varis J, Verberk WJ, Wakefield BJ, McManus RJ (2017). Self-monitoring of blood pressure in hypertension: a systematic review and individual patient data meta-analysis. PLoS Med.

[23] Persell SD, Karmali KN, Stein N, Li J, Peprah YA, Lipiszko D, Ciolino JD, Sato H (2018). Design of a randomized controlled trial comparing a mobile phone-based hypertension health coaching application to home blood pressure monitoring alone: the Smart Hypertension Control Study. Contemp Clin Trials.

[24] Xu H, Long H (2020). The effect of smartphone app-based interventions for patients with hypertension: systematic review and meta-analysis. JMIR Mhealth Uhealth.

[25] Mileski M, Kruse CS, Catalani J, Haderer T (2017). Adopting telemedicine for the self-management of hypertension: systematic review. JMIR Med Inform.

[26] Logan AG, McIsaac WJ, Tisler A, Irvine MJ, Saunders A, Dunai A, Rizo CA, Feig DS, Hamill M, Trudel M, Cafazzo JA (2007). Mobile phone-based remote patient monitoring system for management of hypertension in diabetic patients. Am J Hypertens.

[27] Thangada ND, Garg N, Pandey A, Kumar N (2018). The emerging role of mobile-health applications in the management of hypertension. Curr Cardiol Rep.

[28] Márquez Contreras E, de la Figuera von Wichmann M, Gil Guillén V, Ylla-Catalá A, Figueras M, Balaña M, Naval J (2004). [Effectiveness of an intervention to provide information to patients with hypertension as short text messages and reminders sent to their mobile phone (HTA-Alert)]. Aten Primaria.

[29] Elliott RA, Shinogle JA, Peele P, Bhosle M, Hughes DA (2008). Understanding medication compliance and persistence from an economics perspective. Value Health.

[30] Chapman GB, Brewer NT, Coups EJ, Brownlee S, Leventhal H, Leventhal EA (2001). Value for the future and preventive health behavior. J Exp Psychol Appl.

[31] DeFulio A, Silverman K (2012). The use of incentives to reinforce medication adherence. Prev Med.

[32] Giuffrida A, Torgerson DJ (1997). Should we pay the patient? review of financial incentives to enhance patient compliance. BMJ.

[33] Johnston M, Sniehotta F (2010). Financial incentives to change patient behaviour. J Health Serv Res Policy.

[34] Volpp KG, John LK, Troxel AB, Norton L, Fassbender J, Loewenstein G (2008). Financial incentive-based approaches for weight loss: a randomized trial. JAMA.

[35] Eysenbach G, CONSORT-EHEALTH Group (2011). CONSORT-EHEALTH: improving and standardizing evaluation reports of Web-based and mobile health interventions. J Med Internet Res.

[36] Lacruz ME, Kluttig A, Kuss O, Tiller D, Medenwald D, Nuding S, Greiser KH, Frantz S, Haerting J (2017). Short-term blood pressure variability - variation between arm side, body position and successive measurements: a population-based cohort study. BMC Cardiovasc Disord.

[37] Levey Andrew S, Stevens Lesley A, Schmid Christopher H, Zhang Yaping Lucy, Castro Alejandro F, Feldman Harold I, Kusek John W, Eggers Paul, Van Lente Frederick, Greene Tom, Coresh Josef, CKD-EPI (Chronic Kidney Disease Epidemiology Collaboration) (2009). A new equation to estimate glomerular filtration rate. Ann Intern Med.

[38] Agarwal R, Light RP (2010). The effect of measuring ambulatory blood pressure on nighttime sleep and daytime activity--implications for dipping. Clin J Am Soc Nephrol.

[39] O'Brien E, Parati G, Stergiou G (2013). Ambulatory blood pressure measurement: what is the international consensus?. Hypertension.

[40] Parati G, Stergiou GS, Asmar R, Bilo G, de LP, Imai Y, Kario K, Lurbe E, Manolis A, Mengden T, O'Brien E, Ohkubo T, Padfield P, Palatini P, Pickering TG, Redon J, Revera M, Ruilope LM, Shennan A, Staessen JA, Tisler A, Waeber B, Zanchetti A, Mancia G (2010). European Society of Hypertension practice guidelines for home blood pressure monitoring. J Hum Hypertens.

[41] Daskalopoulou SS, Rabi DM, Zarnke KB, Dasgupta K, Nerenberg K, Cloutier L, Gelfer M, Lamarre-Cliche M, Milot A, Bolli P, McKay DW, Tremblay G, McLean D, Tobe SW, Ruzicka M, Burns KD, Vallée M, Ramesh PGV, Lebel M, Feldman RD, Selby P, Pipe A, Schiffrin EL, McFarlane PA, Oh P, Hegele RA, Khara M, Wilson TW, Brian PS, Burgess E, Herman RJ, Bacon SL, Rabkin SW, Gilbert RE, Campbell TS, Grover S, Honos G, Lindsay P, Hill MD, Coutts SB, Gubitz G, Campbell NRC, Moe GW, Howlett JG, Boulanger J, Prebtani A, Larochelle P, Leiter LA, Jones C, Ogilvie RI, Woo V, Kaczorowski J, Trudeau L, Petrella RJ, Hiremath S, Stone JA, Drouin D, Lavoie KL, Hamet P, Fodor G, Grégoire JC, Fournier A, Lewanczuk R, Dresser GK, Sharma M, Reid D, Benoit G, Feber J, Harris KC, Poirier L, Padwal RS (2015). The 2015 Canadian Hypertension Education Program recommendations for blood pressure measurement, diagnosis, assessment of risk, prevention, and treatment of hypertension. Can J Cardiol.

[42] Guo W, Li B, He Y, Xue Y, Wang H, Zheng Q, Xiang D (2014). Validation of the Andon KD-5917 automatic upper arm blood pressure monitor, for clinic use and self-measurement, according to the European Society of Hypertension International Protocol revision 2010. Blood Press Monit.

[43] Parati G, Omboni S, Albini F, Piantoni L, Giuliano A, Revera M, Illyes M, Mancia G, TeleBPCare Study Group (2009). Home blood pressure telemonitoring improves hypertension control in general practice. The TeleBPCare study. J Hypertens.

[44] James PA, Oparil S, Carter BL, Cushman WC, Dennison-Himmelfarb C, Handler J, Lackland DT, LeFevre ML, MacKenzie TD, Ogedegbe O, Smith SC, Svetkey LP, Taler SJ, Townsend RR, Wright JT, Narva AS, Ortiz E (2014). 2014 evidence-based guideline for the management of high blood pressure in adults: report from the panel members appointed to the Eighth Joint National Committee (JNC 8). JAMA.

[45] Lewington S, Clarke R, Qizilbash N, Peto R, Collins R, Prospective SC (2002). Age-specific relevance of usual blood pressure to vascular mortality: a meta-analysis of individual data for one million adults in 61 prospective studies. Lancet.

[46] Rapsomaniki E, Timmis A, George J, Pujades-Rodriguez M, Shah AD, Denaxas S, White IR, Caulfield MJ, Deanfield JE, Smeeth L, Williams B, Hingorani A, Hemingway H (2014). Blood pressure and incidence of twelve cardiovascular diseases: lifetime risks, healthy life-years lost, and age-specific associations in 1·25 million people. Lancet.

[47] Muntner P, Shimbo D, Carey RM, Charleston JB, Gaillard T, Misra S, Myers MG, Ogedegbe G, Schwartz JE, Townsend RR, Urbina EM, Viera AJ, White WB, Wright JT (2019). Measurement of blood pressure in humans: a scientific statement from the American Heart Association. Hypertension.

[48] McEvoy JW, Daya N, Rahman F, Hoogeveen RC, Blumenthal RS, Shah AM, Ballantyne CM, Coresh J, Selvin E (2020). Association of isolated diastolic hypertension as defined by the 2017 ACC/AHA blood pressure guideline with incident cardiovascular outcomes. JAMA.

[49] Franklin SS, Pio JR, Wong ND, Larson MG, Leip EP, Vasan RS, Levy D (2005). Predictors of new-onset diastolic and systolic hypertension: the Framingham Heart Study. Circulation.

[50] Yano Y, Stamler J, Garside DB, Daviglus ML, Franklin SS, Carnethon MR, Liu K, Greenland P, Lloyd-Jones DM (2015). Isolated systolic hypertension in young and middle-aged adults and 31-year risk for cardiovascular mortality: the Chicago Heart Association Detection Project in Industry study. J Am Coll Cardiol.

[51] Li Y, Wei F, Thijs L, Boggia J, Asayama K, Hansen TW, Kikuya M, Björklund-Bodegård K, Ohkubo T, Jeppesen J, Gu Y, Torp-Pedersen C, Dolan E, Liu Y, Kuznetsova T, Stolarz-Skrzypek K, Tikhonoff V, Malyutina S, Casiglia E, Nikitin Y, Lind L, Sandoya E, Kawecka-Jaszcz K, Mena L, Maestre GE, Filipovský J, Imai Y, O'Brien E, Wang J, Staessen JA, International Database on Ambulatory blood pressure in relation to Cardiovascular Outcomes (IDACO) Investigators (2014). Ambulatory hypertension subtypes and 24-hour systolic and diastolic blood pressure as distinct outcome predictors in 8341 untreated people recruited from 12 populations. Circulation.

[52] EuroQol G (1990). EuroQol--a new facility for the measurement of health-related quality of life. Health Policy.

[53] Broadbent E, Petrie KJ, Main J, Weinman J (2006). The brief illness perception questionnaire. J Psychosom Res.

[54] Report of the National Nutrition Survey 2010. Health Promotion Board Singapore.

[55] Bharmal M, Payne K, Atkinson MJ, Desrosiers M, Morisky DE, Gemmen E (2009). Validation of an abbreviated Treatment Satisfaction Questionnaire for Medication (TSQM-9) among patients on antihypertensive medications. Health Qual Life Outcomes.

[56] Carrasco MP, Salvador CH, Sagredo PG, Márquez-Montes J, González DMMA, Fragua JA, Rodríguez MC, García-Olmos LM, García-López F, Carrero AM, Monteagudo JL (2008). Impact of patient-general practitioner short-messages-based interaction on the control of hypertension in a follow-up service for low-to-medium risk hypertensive patients: a randomized controlled trial. IEEE Trans Inf Technol Biomed.

