# Supplementary material for: Wireless Home Blood Pressure Monitoring System With Automatic Outcome-Based Feedback and Financial Incentives to Improve Blood Pressure in People With Hypertension: Protocol for a Randomized Controlled Trial
Source: JMIR Res Protoc. 2021 Jun 9;10(6):e27496. doi: 10.2196/27496 (PMC8262550; doi:10.2196/27496)
Supplement: Multimedia Appendix 2 [file resprot_v10i6e27496_app2.pdf]

SingHealth

PARTICIPANT INFORMATION SHEET AND CONSENT FORM

STUDY INFORMATION

Protocol Title:

A randomised controlled trial to improve hypertension outcomes using wireless home blood pressure monitoring with automatic outcome-based feedback and financial incentives

Simplified title: WIreless monitoring and Financial incentives for uncontrolled HYpertension (WIFHY) study

Site Principal Investigator:

Dr XXX
SingHealth Polyclinics – Bedok
Heartbeat@Bedok
11 Bedok North Street 1
#02-01 / #03-01
Singapore 469662
Phone No.: (65) 6XXX XXXX

Site Co-Investigators:

Dr XXX
SingHealth Polyclinics – Bedok
Heartbeat@Bedok
11 Bedok North Street 1
#02-01 / #03-01
Singapore 469662
Phone No.: (65) 6XXX XXXX

Dr XXX
SingHealth Polyclinics – Bedok
Heartbeat@Bedok
11 Bedok North Street 1
#02-01 / #03-01
Singapore 469662
Phone No.: (65) 6XXX XXXX

OR

Site Principal Investigator:

Dr XXX
SingHealth Polyclinics – Marine Parade
Blk 80,
Marine Parade Central,
#01-792
Singapore 440080
Phone No.: (65) 6XXX XXXX

Site Co-Investigators:

Dr XXX
SingHealth Polyclinics – Marine Parade
Blk 80,
Marine Parade Central,
#01-792
Singapore 440080
Phone No.: (65) 6XXX XXXX

Dr XXX
SingHealth Polyclinics – Marine Parade
Blk 80,
Marine Parade Central,
#01-792
Singapore 440080
Phone No.: (65) 6XXX XXXX

Sponsor:

Ministry of Health National Medical Research Council (MOH NMRC)

PURPOSE OF THE RESEARCH STUDY

You are being invited to participate in a research study. Before you take part in this research study, the study must be explained to you and you must be given the chance to ask questions. Please read carefully the information provided here. If you agree to participate, please sign the consent form. You will be given a copy of this document to take home with you.

This study involves wireless home blood pressure monitoring and financial incentives for uncontrolled hypertension. We hope to learn whether a wireless home blood pressure monitoring system with and without financial incentives is effective at reducing blood pressure (BP) compared to usual care in the Polyclinic that relies on patient self-reporting and best practices.

You were selected as a possible participant in this study because:

- You are on anti-hypertensive medication
- Your blood pressure remains high

This study will recruit 224 participants from Bedok Polyclinic and Marine Parade Polyclinic.

STUDY PROCEDURES AND VISIT SCHEDULE

If you agree to take part in this study, you will be asked to put on an Ambulatory Blood Pressure Monitor at baseline (i.e. when study starts) and at the Month 6 (Week 24) visit. You will also be asked to measure your blood pressure at home using one of the study's home blood pressure monitors. Depending on the study group you are allocated to, you will use a wireless or non-wireless blood pressure monitor.

Your adherence to your hypertensive medications will be monitored using an electronic medication container called the eCAP™. The eCAP™ is an electronic cap that goes over selected medication containers. It records the date and time you open the medication container and stores this information in its memory. The recorded information would then be downloaded and transferred to a computer. The electronic medication container and blood pressure monitor will be loaned to you and will be collected from you at the end of the study.

If you agree to take part in this study, you will be randomised to 1 of 3 study groups. Randomisation means assigning you to one of 3 groups by chance, like tossing a coin or rolling a dice. Neither you nor the study staff will be able to decide or change your group allocation. The study groups are:

1. Group 1, Usual Care: Participants in the usual care group will use an automatic (non-wireless) blood pressure monitor commonly available at the Polyclinic. You would be given an automatic (non-wireless) blood pressure monitor if you do not have one. The self-monitoring instructions are adapted from the guidelines from the Healthy Singapore or healthy.sg website.
2. Group 2, Wireless Home Blood Pressure Monitoring: Participants in this group will use a wireless blood pressure monitor and a smartphone application to monitor their home blood pressure. The monitor will transmit information to the participants' smartphone via Bluetooth. Readings will be displayed on a smartphone application and the data will be automatically sent to a secure trial website. The trial website will then send feedback messages via SMS to the participants' smartphone and automatically trigger interventions from the Polyclinic, if need be, depending on the patient's blood pressure reading(s).
3. Group 3, Wireless Home Blood Pressure Monitoring with Incentives: Participants in this group will receive an intervention identical to that in Group 2, with the addition of modest financial incentives for monitoring their BP. This group is subdivided into two sub-groups (Instant Reward Sub-group and Health Capital Sub-group) who are eligible to receive the same incentive amounts but framed differently. In both subgroups, participants can receive incentives worth up to $ 216 at the end of the study, for monitoring their BP at least 3 times per week.

   Group 3a (Instant Reward): In the Instant Reward Sub-group, patients receives $ 3 for each day he/she measures his/her BP, up to three times per week. There are no financial incentives if participants do not measure their BP.

   Group 3b (Health Capital): *Health capital is an incentive amount* that increases when you measure your BP as recommended and decreases when you miss measurements. In this sub-group, each patient receives an initial starting amount of $72. Each patient earns an additional $6 on each week where he/she measures his/her BP on at least 3 different days; however the earned amount decreases weekly by 10% per missing BP reading.

The table illustrates the scheme:

	**Group 3a: Instant Reward**	**Group 3b: Health Capital**
Starting amount	$0	$72
Days with BP measures in the week		
3 or more days	$ 9 earned	▲ $6 higher
2 days	$ 6 earned	▼ 10% lower
1 day	$ 3 earned	▼▼ 20% lower
0 days	$ 0 earned	▼▼▼ 30% lower

Maximum total earnings (after 24 weeks)	$216	$216

The tables below give an example on how the Instant Reward and Health Capital incentive subgroups work:

Example 1: Group 3a- Instant Reward subgroup

Week	Days with BP measurement(s)	Weekly incentive	Cumulative Earnings
Starting amount	--	--	**$0**
Week 1	3 days	$9 earned	0 + 9 = **$9**
Week 2	1 day	$3 earned	9 + 3 = **$12**
Week 3	2 days	$6 earned	12 + 6 = **$18**
Week 4	0 days	$0 earned	18 + 0 = **$18**
…	…	…	…
Week 24	…	…	…

Example 2: Group 3b- Health Capital subgroup

Week	Days with BP measurement(s)	Weekly change in health capital	Health Capital
Starting amount	--	--	**$72.00**
Week 1	3 days	▲ $6 higher	72+ 6 = **$78**
Week 2	2 days	▼ 10% lower	78– 8 = **$70**
Week 3	1 day	▼▼ 20% lower	70– 14 = **$56**
Week 4	0 day	▼▼▼ 30% lower	56– 17 = **$39**
…	…	…	…
Week 24	…	…	…

Note: For participants in Arm 3b, the amount earned or lost for the week and the total amount earned till present will be rounded off to the nearest dollar (e.g. $39.31 will be rounded off to $39).

Your participation in the study will last for 6 months (24 weeks). You will use the blood pressure monitor for at least 3 times in a week for the entire study and be followed up for the 6-month

(24-week) duration of the study. You will need to visit the polyclinic at least 2 times in the course of the study.

Schedule of visits and procedures:

If you agree to take part in this study, you will be asked to attend at least 2 study visits: 1 at baseline (i.e. when study starts) and 1 at the end of the study (Month 6). Your doctor will see you at baseline, during the study intervention (as advised by the doctor) and at Month 6 for standard medical care. Participants from Groups 2 and 3 who have been monitored to have controlled BP for at least 28 days are potential candidates for skipping the clinic visits that fall between the baseline visit and Month 6 visit. The decision to skip any clinic visit is by the doctor after other clinical factors have been considered (e.g. diabetes control). At baseline and at the end of the study (Month 6), questionnaires/ checklists will be administered to you and study devices will be managed by the clinical research coordinator. Your participation in the study will last at least 6 months.

	Doctor's consultation	Questionnaires and Checklists	Study devices issued / data downloaded (if applicable)	Incentives paid* (if applicable)
Baseline visit	✓	✓	✓	✓
Follow-up clinic visits	✓ *except for participants in Groups 2 and 3 who have been monitored to have controlled BP for at least 28 days; decision to skip any clinic visit is by the doctor after other clinical factors have been considered (e.g. diabetes control)	X	X	X
Month 6 follow-up visit	✓	✓	✓	✓

Additional Study Procedures

Any individually-identifiable data obtained during the course of this study will be stored and used only for the purposes of this study. These data will not be used for future research. Information on your medical record or pharmacy claims relating to your hypertension such as the number of anti-hypertensive medications, dose frequency and number of prescription refills may also be retrieved and used for analyses. All such personal medical information will be de-identified at the earliest opportunity. De-identification is the process where the study team will mask your personal identifiers, such as your name and NRIC, to protect your privacy.

All medical information will only be used by the study team for the purpose of this study. The study checklists and questionnaires, including the Screener, will be used by the study team for research purpose. These questionnaire or checklists will gather information on your medication beliefs, health status, physical activity, dietary practices, list of medications, height, weight and blood pressure readings.

When your participation in the study ends, you will no longer have access to the study devices (i.e., Ambulatory blood pressure monitor ,home blood pressure monitor, electronic medication container), and wireless home blood pressure monitoring system and financial incentives (if applicable).

YOUR RESPONSIBILITIES IN THIS STUDY

If you agree to participate in this study, you should*:*

- Use the study devices as instructed and follow the advice given to you by the study team.
    - Measure your blood pressure using the automatic (non-wireless) blood pressure monitor (Group 1) or the wireless blood pressure monitor (Groups 2 and 3) at least 3 times a week.
    - The medication container should only be opened when you take your medicine(s), and shut thereafter
    - Measure your blood pressure using an ambulatory blood pressure monitor at 2 study visits, at baseline and at the end of the study.
- Be prepared to visit Bedok/ Marine Parade Polyclinic at least 2 times and undergo all the procedures that are outlined above. Care will be taken to coordinate study visits with scheduled clinic visits to minimize participation burden.
- Keep to your study appointments. If it is necessary to miss an appointment, please contact the Clinical Research Coordinator at XXXX XXXX to reschedule as soon as you know you will miss the appointment.
- Inform the Site Principal Investigator as soon as possible about any side effects that you may have encountered.

WHAT IS NOT STANDARD CARE OR EXPERIMENTAL IN THIS STUDY

The study is being conducted because the use of financial incentives, wireless home blood pressure monitoring and the use of the eCAP™ are not used as part of the usual care in participants with uncontrolled hypertension. We hope that your participation will help us to determine whether a wireless home blood pressure monitoring system with and without financial incentives is effective at reducing blood pressure compared to usual care in the Polyclinic that relies on patient self-reporting and best practices. Please note that financial incentives are offered as part of the study, and will only be used throughout the study duration. Randomization is only done for research studies.

Although home blood pressure monitoring and health counselling may be part of standard medical care, in this study these procedures are being performed for the purposes of the research.

POSSIBLE RISKS, DISCOMFORTS AND INCONVENIENCES

The basic component of this study, BP self-monitoring with automatic messages based on submitted BP readings, is modelled after an existing standard of care protocol by the Polyclinic. Any changes to your medications will be reviewed by your polyclinic and the Site Clinical Research Coordinator will verify BP readings before cancelling any upcoming visit.

This study does not involve drugs in addition to what is prescribed in your usual care. Investigators and study staff will be trained in the use of study devices and storage through careful reading and preparation based on user guides and materials provided by the device manufacturers. Nevertheless, some side effects (e.g. discomfort, bruising and skin irritation)

may occur with the use of the Ambulatory Blood Pressure Monitor. Note that you will be expected to wear the Ambulatory Blood Pressure Monitor for 12 hours at the beginning and at the end of the study. For these reasons, this study is deemed to be of low risk by the investigators, and involves no greater risks than that would occur for an individual on a typical care schedule.

POTENTIAL BENEFITS

If you participate in this study you may reasonably expect to benefit from the study intervention. Ambulatory blood pressure monitoring will provide information on your blood pressure throughout a 12 hour period. This would assist doctors in evaluating the efficacy of your treatment. Home blood pressure monitoring will provide you with the skills and knowledge to manage hypertension. Better management of hypertension and blood pressure outcomes are expected as a result of your participation in this study, to the extent that you respond and adhere to the instructions given. Additionally, your participation will also contribute to the medical knowledge regarding the use and cost-effectiveness of using financial incentives and wireless home blood pressure monitoring systems on clinical outcomes and adherence behaviour. You may also earn incentives for your participation.

ALTERNATIVES

If you choose not to take part in this study, the alternative is to have what is considered standard care for your condition. In our institution this would be home blood pressure monitoring and assessment of your condition during your regular visit to your physician.

COSTS OF PARTICIPATION

If you take part in this study, the following will be performed at no charge to you:

- Health counselling on home blood pressure monitoring
- Ambulatory Blood Pressure monitoring
- Use of blood pressure monitors and electronic medication container, eCAP™

If you take part in this study, you will have to pay for the following:

- Costs incurred as part of your usual care schedule such as for doctor consultations and hypertensive medications

You will be reimbursed for your time, inconvenience, telecommunication and transportation costs as follows:

<table>
<tr><th></th><th>Group 1</th><th>Group 2</th><th>Group 3</th><th>To be issued on</th></tr>
<tr><td>Completion of baseline assessment</td><td>$ 25</td><td>$ 25</td><td>$ 25</td><td>Baseline visit</td></tr>
<tr><td>Attending all study visits</td><td>$25</td><td>$25</td><td>$25</td><td rowspan="3">Month 6 visit</td></tr>
<tr><td>Fairness payment</td><td>$100</td><td>$100</td><td>-</td></tr>
<tr><td>Adherence to blood pressure monitoring</td><td>-</td><td>-</td><td>Varies (up to $216,</td></tr>
</table>

			see page 2 for more details)	
Telecommunication costs	-	$30	$30	

Payments will be made in the form of NTUC vouchers at study visits.

INCIDENTAL FINDINGS

In the case of an "incidental finding" (i.e. any abnormality that we did not expect to see in this study or unrelated to the purpose of this study), we will not re-identify and give you any results from the research.

PARTICIPANT'S RIGHTS

Your participation in this study is entirely voluntary. Your questions will be answered clearly and to your satisfaction.

In the event of any new information becoming available that may be relevant to your willingness to continue in this study, you will be informed in a timely manner by the Site Principal Investigator or his/her representative and will be contacted for further consent if required.

By signing and participating in the study, you do not waive any of your legal rights to revoke your consent and withdraw from the study at any time.

WITHDRAWAL FROM STUDY

You are free to withdraw your consent and discontinue your participation at any time without prejudice to you or effect on your medical care. If you decide to stop taking part in this study, you should tell the Site Principal Investigator.

If you withdraw from the study,

- Please contact the Site Clinical Research Coordinator or Site Principal Investigator.
- You will be asked to return all study devices (blood pressure monitors, eCAP) to the study team. This should be arranged with the Site Clinical Research Coordinator.
- A short exit questionnaire and checklist will be administered to take note of the reason(s) of withdrawal.

However, the data that have been collected until the time of your withdrawal will be kept and analysed. The reason is to enable a complete and comprehensive evaluation of the study.

Your doctor, the Site Principal Investigator and/or the Sponsor of this study may stop your participation in the study at any time for one or more of the following reasons*:*

- Failure to follow the instructions of the Site Clinical Research Coordinator or Site Principal Investigator.
- The Site Principal Investigator decides that continuing your participation could be harmful.
- Pregnancy (if applicable.)
- It is possible that, during the course of the 6-month intervention, your condition is deemed unsatisfactory by your doctor (typically during one of the follow-up visits). At this point, you may be ineligible for the study. The Site Study Coordinator takes note of the reason for

ineligibility and proceeds with payment of SGD 80 in compensation for forgoing potential payments that you might have received had you remained in the study.

- The study is cancelled.

RESEARCH RELATED INJURY AND COMPENSATION

If you follow the directions of the Principal Investigator (Investigators and/ or Clinical Research Coordinator) of this research study and you are injured due to the research procedure given under the plan for the research study, our institution will provide you with the appropriate medical treatment.

Payment for management of the normally expected consequences of your treatment will not be provided by SingHealth Polyclinics.

You still have all your legal rights. Nothing said here about treatment or compensation in any way alters your right to recover damages where you can prove negligence.

CONFIDENTIALITY OF STUDY AND MEDICAL RECORDS

Your participation in this study will involve the collection of Personal Data. Personal Data collected for this study will be kept confidential. Your records, to the extent of the applicable laws and regulations, will not be made publicly available. Only your Investigator(s) will have access to the confidential information being collected.

However, the Sponsor *(MOH NMRC)*, Regulatory Agencies, Institutional Review Board and Ministry of Health will be granted direct access to your original medical records to check study procedures and data, without making any of your information public.

By signing the Consent Form, you consent to (i) the collection, access to, use and storage of your Personal Data by SingHealth Polyclinics – Bedok/ Marine Parade Polyclinic, and (ii) the disclosure of such Personal Data to our authorised service providers and relevant third parties.

"Personal Data" means data about you which makes you identifiable (i) from such data or (ii) from that data and other information which an organisation has or likely to have access. Examples of personal data include medical conditions, medications, investigations and treatment history.

Research arising in the future, based on this "Personal Data", will be subject to review by the relevant institutional review board.

By participating in this research study, you are confirming that you have read, understood and consent to the SingHealth Data Protection Policy, the full version of which is available at www.singhealth.com.sg/pdpa. Hard copies are also available on request.

Data collected and entered into the study checklists and questionnaires are the property of Duke-NUS Medical School Singapore and SingHealth Polyclinics. In the event of any publication regarding this study, your identity will remain confidential.

WHO TO CONTACT IF YOU HAVE QUESTIONS REGARDING THE STUDY

If you have questions about this research study or in the case of any injuries during the course of this study, you may contact the Clinical Research Coordinator at XXXX XXXX or the Site Principal Investigator Dr XXX at Tel: (65) 6XXX XXXX.

WHO HAS REVIEWED THE STUDY

This study has been reviewed by the SingHealth Centralised Institutional Review Board for ethics approval.

If you have questions about your rights as a participant, you can call the SingHealth Centralised Institutional Review Board at 6XXX XXXX during office hours (8:30 am to 5:30pm).

If you have any feedback about this research study, you may contact the Site Principal Investigator or the SingHealth Centralised Institutional Review Board.

CONSENT FORM
**Details of Research Study**
**Protocol Title:**<br>A randomised controlled trial to improve hypertension outcomes using wireless home blood pressure monitoring with automatic outcome-based feedback and financial incentives<br><br>**Simplified title:** WIreless monitoring and Financial incentives for uncontrolled HYpertension (WIFHY) study<br><br>**Site Principal Investigator:** Dr XXX, SingHealth Polyclinics – Bedok / Marine Parade Polyclinic, Heartbeat@Bedok, 11 Bedok North Street 1, #02-01/ #03-01, Singapore 469662. OR Blk 80, Marine Parade Central, #01-792, Singapore 440080. Tel: (65) 6XXX XXXX
I agree to participate in the research study as described and on the terms set out in the Participant Information Sheet.<br><br>I have fully discussed and understood the purpose and procedures of this study. I have been given the Participant Information Sheet and the opportunity to ask questions about this study and have received satisfactory answers and information.<br><br>I understand that my participation is voluntary and that I am free to withdraw at any time, without giving any reasons and without my medical care being affected.<br><br>By participating in this research study, I confirm that I have read, understood and consent to the SingHealth Data Protection Policy.<br><br>Name of participant ____ Signature/Thumbprint (Right / Left) ____ Date of signing ____

To be completed by parent / legal guardian / legal representative, where applicable

I hereby give consent for the above participant to participate in the proposed research study. The nature, risks and benefits of the study have been explained clearly to me and I fully understand them.

I confirm that I have read, understood and consent to the SingHealth Data Protection Policy.

_______________________ _______________________ _______________

Name of participant's parent/ legal guardian/ legal representative | Signature/ Thumbprint (Right / Left) | Date of signing

To be completed by translator, if required

The study has been explained to the participant/ legal representative in

_______________________ by _______________________.

Language | Name of translator

To be completed by witness, where applicable

I, the undersigned, certify that:

- I am 21 years of age or older.
- To the best of my knowledge, the participant or the participant's legal representative signing this informed consent form had the study fully explained in a language understood by him/ her and clearly understands the nature, risks and benefits of his/ her participation in the study.
- I have taken reasonable steps to ascertain the identity of the participant or the participant's legal representative giving the consent.
- I have taken steps to ascertain that the consent has been given voluntarily without any coercion or intimidation.

Witnessed by: _______________________ _______________________

Name of witness | Date of signing

____

Signature of witness

1. An impartial witness (who is 21 years of age or older, has mental capacity, who is independent of the research study, and cannot be unfairly influenced by people involved with the research study) should be present during the entire informed consent discussion if a participant or the participant's legal representative is unable to read, and/or sign and date on the consent form (i.e. using the participant or legal representative thumbprint). After the written consent form and any written information to be provided to participant, is read and explained to the participant or the participant's legal representative, and after the participant or the participant's legal representative has orally consented to the participant's participation in the study and, if capable of doing so, has signed and personally dated the consent form, the witness should sign and personally date the consent form. This is applicable for Clinical Trials regulated by HSA and Human Biomedical Research under HBRA.

2. For HBRA studies, the witness may be a member of the team carrying out the research only if a participant or the participant's legal representative is able to read, sign and date on the consent form.

Investigator's Statement

I, the undersigned, certify to the best of my knowledge that the participant/ participant's legal representative signing this consent form had the study fully explained and clearly understands the nature, risks and benefits of his/ her/ his ward's/ her ward's participation in the study.

______________________________	______________________________	____________________
Name of Investigator/<br>Clinical Research Coordinator	Signature	Date

ANNEX A

The Prescription In Locker Box (PILBOX) is 24-hour service offered by SingHealth Polyclinics that allows you or your caregiver to collect your repeat prescription at your convenience, without having to queue at the pharmacy.

The PILBOX service is situated outside Bedok/ Marine Parade Polyclinic.

You will be informed by the Clinical Research Coordinator if you are eligible to collect your medication through the PILBOX. You are eligible to collect your medication through PILBOX if you have shown to have good blood pressure control over the course of 28 days and if there are no changes to be made to your medication.

By acknowledging this section, you understand and agree that no medication counselling will be provided for the medication that is to be collected from the PILBOX. The medication is to be collected within 24 hours of receiving the SMS notification that it is ready for collection. You also agree to complete payment for the medication that is to be collected from the PILBOX.

Your consent does not preclude your participation in this study.
Your utilisation of PILBOX service need not be continuous. You can choose to collect your next round of medication through the pharmacy staff as PILBOX service is per prescription basis.

Name of participant: ____________________________________

______________________________________ ______________________

Signature/Thumbprint (Right / Left) of participant Date of signing

*Please note that medication(s) supplied/collected are non-exchangeable and non-refundable. Please check that all medication(s) are correct and in good condition upon collection from the PILBOX station. The person collecting the medications must be aged 18 years and above.

For Clinical Research Coordinator to fill up:

- ☐ Participant does not wish to collect medication through PILBOX
