# Supplementary material for: Wireless Home Blood Pressure Monitoring System With Automatic Outcome-Based Feedback and Financial Incentives to Improve Blood Pressure in People With Hypertension: Protocol for a Randomized Controlled Trial
Source: JMIR Res Protoc. 2021 Jun 9;10(6):e27496. doi: 10.2196/27496 (PMC8262550; doi:10.2196/27496)
Supplement: Multimedia Appendix 3 [file resprot_v10i6e27496_app3.pdf]

Multimedia Appendix 3: ABPM
-Figure S1: ABPM Diary

WIFHY Ambulatory Blood Pressure Monitor (ABPM) Diary

1) ABPM information [*To be filled up by Clinical Research Coordinator (CRC*)]

PID no:_____<br>Study Site: Bedok/ Marine Parade<br>Type of test: Baseline/ Month 6	ABPM information<br>1) S/N:__________________<br>2) Cuff size: Child/ Adult/ Large Adult<br>3) Arm used: Right/ Left
ABPM was started on:<br>*Date:____________(dd/mm/yyyy)*<br>*Time:____________(hh:mm)*	ABPM is to be returned to CRC on: *Date:_________(dd/mm/yyyy)*<br>*Time:____________(hh:mm)*

2) Blood Pressure (BP) Medication Diary (*To be filled up by participant*)

Time	Medication name	Medication dose	Remarks
e.g.7:00pm	*Atenolol*	*50mg*	*Took medicine after dinner*

3) Activity Diary (*To be filled up by participant*)

Time	Activity
e.g.1:00pm-1:30pm	*e.g. Was brisk walking to food court*

Thank you for your participation in the WiFHy study!

-Figure S2: Workflow for ABPM results review at Baseline

1) CRC notes the following parameters:
a) Average daytime SBP and DBP
b) The percentage of abnormal readings.
c) The minimum and maximum SBP and DBP.

↓

2) CRC highlights abnormal average day time ABPM readings and classifies based on the study's BP classification (i.e. Very Low/ Very High/ Extremely High) for review by the study doctor.

↓

3) The study doctor will review the test results within 3 working days of the test result being available (after the participant returns the ABPM and the test result has been extracted). The study doctor documents in the participant's medical records for any interventions carried out.

Some recommendations for intervening:

a) For Very Low BP:
   - Check for postural dizziness, breathlessness.
   - Check for dehydration/ bleeding e.g. Per rectum.
   - Consider down-titrating the anti-hypertensive medications.
   Review within 2-4 weeks.

b) Very High BP (Grade 2 Hypertension):
   - Consider adding 2 anti-hypertensive medications, or titrate 1, and adding another anti-hypertensive medication.

c) Extremely high (Grade 3 Hypertension)
   - Checks for target organ damage (i.e. chest pain, breathlessness, new stroke, worsening renal function, ECG with changes). To A&E if symptomatic, if not to start 2 anti-hypertensive medications and review in 2-3 days.

Note: The ABPM test results will be scanned into the patient's medical records as per SHP's workflow for storage of test results.

-Figure S3: Workflow for ABPM results review at Month 6

1) CRC notes the following parameters:
a) Average daytime SBP and DBP
b) The percentage of abnormal readings.
c) The minimum and maximum SBP and DBP.

↓

2) CRC highlights abnormal average day time ABPM readings and classifies based on the study's BP classification (i.e. Very Low/ Very High/ Extremely High) for review by the study doctor.

↓

3) The study doctor will review the test results within 3 working days of the test result being available (after the participant returns the ABPM and the test result has been extracted). The study doctor documents in the participant's medical records for any interventions carried out.

Some recommendations for intervening:

a) For Very Low BP:
   - Check for postural dizziness, breathlessness.
   - Check for dehydration/ bleeding e.g. Per rectum.
   - Consider down-titrating the anti-hypertensive medications.
   Review within 2-4 weeks.

b) Very High BP (Grade 2 Hypertension):
- Consider adding 2 anti-hypertensive medications, or titrate 1, and adding another anti-hypertensive medication.

c) Extremely high (Grade 3 Hypertension)
- Checks for target organ damage (i.e. chest pain, breathlessness, new stroke, worsening renal function, ECG with changes). To A&E if symptomatic.
If not to start 2 anti-hypertensive medications and review in 2-3 days.

↓

4) CRC will collate both baseline and M6 ABPM results for participants' attending doctors to review. Participant to show ABPM results to the doctor they are consulting.

Note: The ABPM test results will be scanned into the patient's medical records as per SHP's workflow for storage of test results.
