# Supplementary material for: Wireless Home Blood Pressure Monitoring System With Automatic Outcome-Based Feedback and Financial Incentives to Improve Blood Pressure in People With Hypertension: Protocol for a Randomized Controlled Trial
Source: JMIR Res Protoc. 2021 Jun 9;10(6):e27496. doi: 10.2196/27496 (PMC8262550; doi:10.2196/27496)

Multimedia Appendix 4: Study timeline for participants

-Figure A3.1: Study timeline for participants

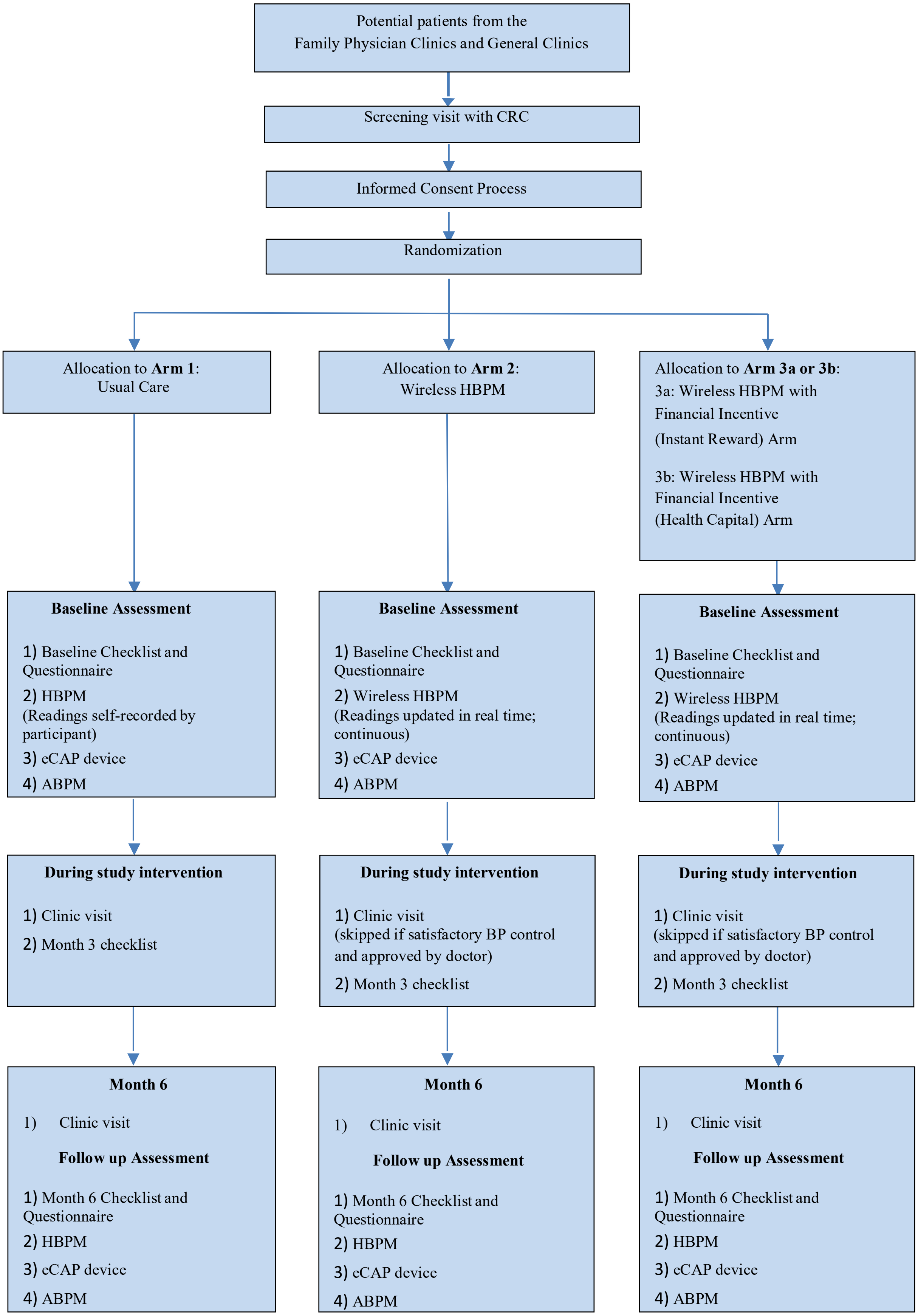

Supplement: Multimedia Appendix 4 [file resprot_v10i6e27496_app4.pdf]
