# Supplementary material for: Wireless Home Blood Pressure Monitoring System With Automatic Outcome-Based Feedback and Financial Incentives to Improve Blood Pressure in People With Hypertension: Protocol for a Randomized Controlled Trial
Source: JMIR Res Protoc. 2021 Jun 9;10(6):e27496. doi: 10.2196/27496 (PMC8262550; doi:10.2196/27496)
Supplement: Multimedia Appendix 5 [file resprot_v10i6e27496_app5.pdf]

Multimedia Appendix 5: Standard guideline for interpretation of BP readings

-Table A5.1: Standard self-monitoring guideline for interpretation of BP readings

**Category**	**Self-monitoring instructions**
Very Low	• Check that the blood pressure monitor is well positioned on your arm and well tightened, and retake your blood pressure reading after 5 minutes of rest.<br>• If your blood pressure is still very low on your 2nd measurement, stop your medicine for blood pressure (if you are on any). See your doctor as soon as possible.
Low Normal	• This blood pressure can be normal in some people, especially if you are on blood pressure medication.<br>• See a doctor immediately if you feel unwell, e.g. postural dizziness, breathless or have chest pain. If you are taking medication, and your blood pressure has mostly been in this range, see your doctor within a few weeks for a review.
Normal	• Continue to monitor your BP as directed.
Slightly High	• Rest for at least 5 minutes, then recheck your blood pressure.<br>• See a doctor immediately if you feel unwell, e.g. postural dizziness, breathless or have chest pain. Otherwise continue to monitor your blood pressure at least 3 times a week. Exercise regularly as tolerated and advised by your doctor.
Very High	• Rest for at least 5 minutes, then recheck your blood pressure.<br>• See a doctor immediately if you feel unwell, e.g. postural dizziness, breathless or have chest pain. Otherwise continue to monitor your blood pressure at least 3 times a week. Reduce your salt intake. Exercise regularly as tolerated and advised by your doctor.
Extremely High	• Check that the blood pressure monitor is well positioned on your arm and well tightened, and retake your blood pressure reading after 5 minutes of rest.<br>• If you feel unwell, e.g. postural dizziness, breathless or have chest pain, call 995 for an ambulance. Otherwise see your doctor as soon as possible.
