# Supplementary material for: Wireless Home Blood Pressure Monitoring System With Automatic Outcome-Based Feedback and Financial Incentives to Improve Blood Pressure in People With Hypertension: Protocol for a Randomized Controlled Trial
Source: JMIR Res Protoc. 2021 Jun 9;10(6):e27496. doi: 10.2196/27496 (PMC8262550; doi:10.2196/27496)

Multimedia Appendix 6: Instant HBPM advice (for Arm 2 and 3 participants)

-Table A6.1: HBPM system action for Instant BP

Category	HBPM System's Actions
Very Low	• On the first occasion, a text message (A1) informing the patient that his/her BP is too low will be sent. The patient will be advised to re-take his/her BP reading within 10 mins.<br>• On the second occasion, if the patient's BP remains very low, a text message (A1.1) will be sent to the patient advising him/her to stop taking the anti-hypertensive medication and to see a doctor as soon as possible. On the study website's dashboard, the system will color code the patient as "Red", which will trigger the CRC to follow the "Red Protocol".<br>• On the second occasion, if the patient's BP is of another category, the system would proceed as per the relevant category (e.g. A1.1, A4.1, A5.1 and A6.1 or A2 and A3).<br>• In the event no BP is taken within 10 mins of the first BP reading, A1.1 will be sent.
Low Normal	• A text message (A2) informing the patient that his/her BP is low normal will be sent.
Normal	• A text message (A3) congratulating the patient on having a BP within range will be sent.
Slightly High	• On the first occasion, a text message (A4) informing the patient that his/her BP is slightly high will be sent. The patient will be advised to re-take his/her BP reading within 10 mins.<br>• On the second occasion, if the patient's BP remains slightly high, a text message (A4.1) would be sent to the patient advising to see a doctor if unwell. Asymptomatic patients are advised to continue BP monitoring and engage in regular exercise.<br>• On the second occasion, if the patient's BP is of another category, the system would proceed as per the relevant category (e.g. A1.1, A4.1, A5.1 and A6.1 or A2 and A3).<br>• In the event no BP is taken within 10 mins of the first BP reading, A4.1 will be sent.
Very High	• On the first occasion, a text message (A5) informing the patient that his/her BP is very high will be sent. The patient will be advised to re-take his/her BP reading within 10 mins.<br>• On the second occasion, if the patient's BP remains very high, a text message (A5.1) would be sent to the patient advising to see a doctor if unwell. Asymptomatic patients are advised to continue BP monitoring, reduce salt intake and engage in regular exercise.<br>• On the second occasion, if the patient's BP is of another category, the system would proceed as per the relevant category (e.g. A1.1, A4.1, A5.1 and A6.1 or A2 and A3).<br>• In the event no BP is taken within 10 mins of the first BP reading, A5.1 will be sent.
Extremely High	• On the first occasion, a text message (A6) informing the patient that his/her BP is extremely high will be sent. The patient will be advised to re-take his/her BP reading within 10 mins.<br>• On the second occasion, if the patient's BP remains extremely high, a text message (A6.1) will be sent informing the patient to contact the polyclinic or call for an ambulance. On the study website's dashboard, the system will color code the patient as "red", which will trigger the CRC to follow the "Red Protocol".<br>• On the second occasion, if the patient's BP is of another category, the system would proceed as per the relevant category (e.g. A1.1, A4.1, A5.1 and A6.1 or A2 and A3).<br>• In the event no BP is taken within 10 mins of the first BP reading, A6.1 will be sent.

-Table A6.2: Instant BP SMSes

Code	SMS Text Message
**A1**	Dear participant, your BP is very low. Check that the BP cuff is well positioned on your arm and well tightened, and retake your BP after 5 mins of rest.
**A1.1**	Dear participant, your BP is very low. Stop medicine for BP. See a Dr ASAP.
**A2**	Dear participant, your BP is low. This BP can be normal, especially if you are on BP medication. See a Dr immediately if you feel unwell, e.g. postural dizziness, breathless or have chest pain. If you are taking medication, and your BP has been in this range, do see your Dr within a few weeks.
**A3**	Dear participant, congratulations! Your BP is normal. Continue to monitor your BP as advised.
**A4**	Dear participant, your BP is slightly high. See a Dr immediately if you feel unwell, e.g. postural dizziness, breathless or have chest pain. Rest for at least 5 mins then recheck your BP.
**A4.1**	Dear participant, your BP is slightly high. See a Dr immediately if you feel unwell, e.g. postural dizziness, breathless or have chest pain. Otherwise continue to monitor your BP at least 3 times a week. Exercise regularly as tolerated and advised by your Dr.
**A5**	Dear participant, your BP is very high. See a Dr immediately if you feel unwell, e.g. postural dizziness, breathless or have chest pain. Rest for at least 5 mins before rechecking your BP.
**A5.1**	Dear participant, your BP is very high. See a Dr immediately if you feel unwell, e.g. postural dizziness, breathless or have chest pain. Otherwise continue to monitor your BP at least 3 times a week. Reduce your salt intake. Exercise regularly as tolerated and advised by your Dr.
**A6**	Dear participant, your BP is extremely high. Check that the BP monitor is well positioned on your arm and well tightened, and retake your BP reading after 5 mins of rest.
**A6.1**	Dear participant, your BP is extremely high. If you feel unwell, e.g. postural dizziness, breathless or have chest pain, call 995 for an ambulance. Otherwise see your Dr ASAP.

-Figure A6.1: Red Protocol (Clinical Protocol for Very Low- and Extremely High- Instant BP)

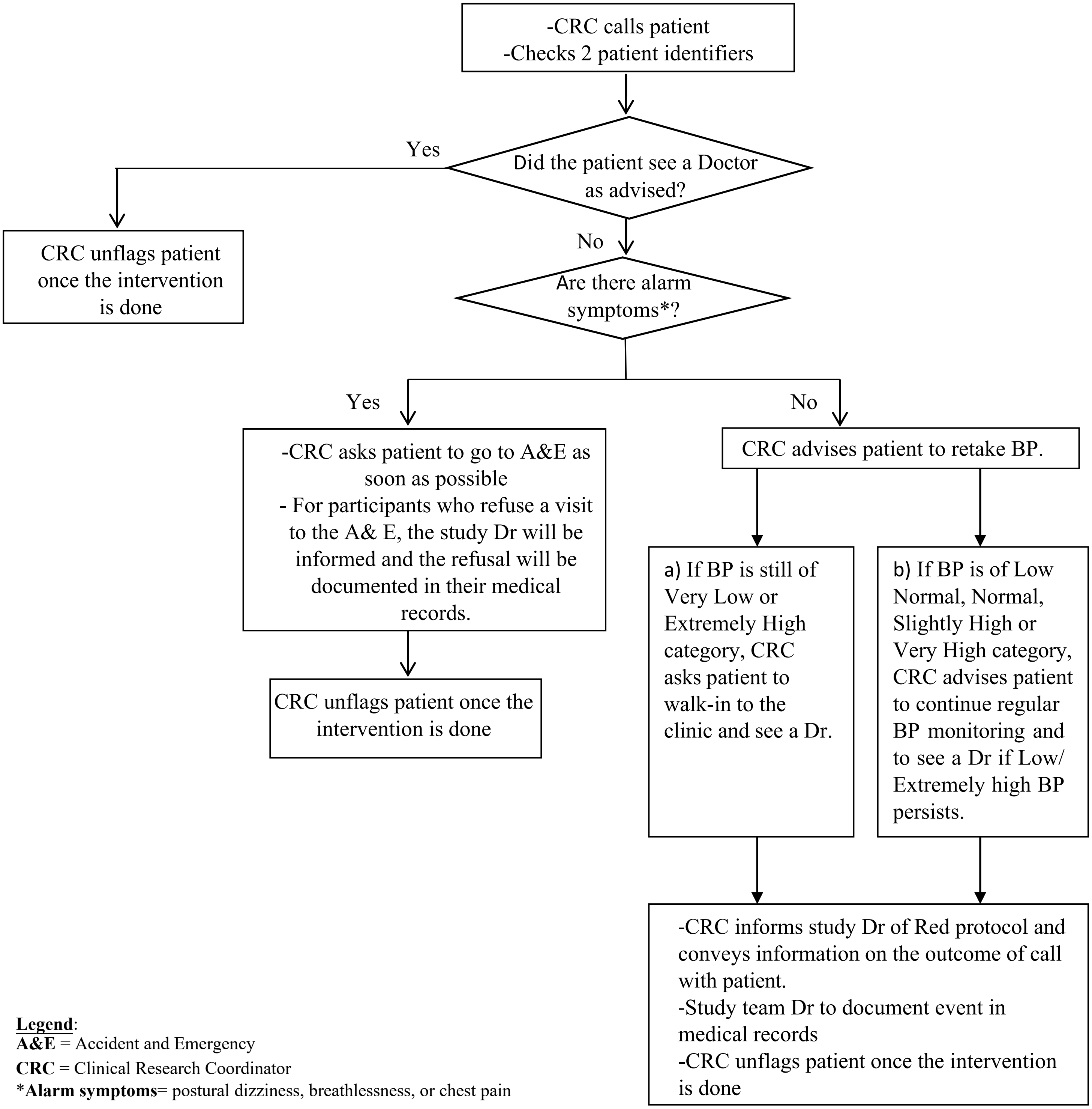

-Figure A6.2: Instant HBPM advice algorithm

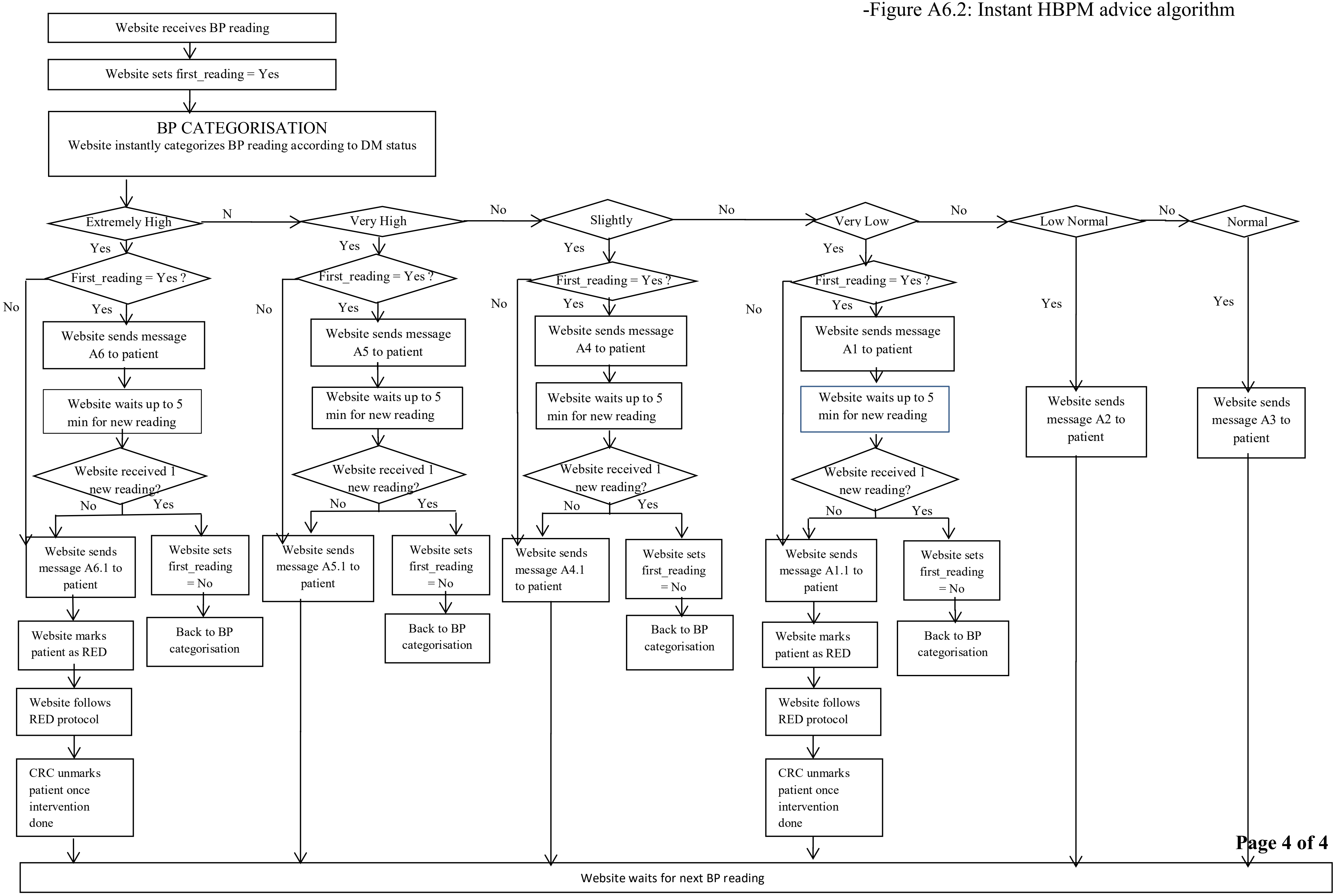

Supplement: Multimedia Appendix 6 [file resprot_v10i6e27496_app6.pdf]
