# Supplementary material for: Wireless Home Blood Pressure Monitoring System With Automatic Outcome-Based Feedback and Financial Incentives to Improve Blood Pressure in People With Hypertension: Protocol for a Randomized Controlled Trial
Source: JMIR Res Protoc. 2021 Jun 9;10(6):e27496. doi: 10.2196/27496 (PMC8262550; doi:10.2196/27496)

Multimedia Appendix 7: Weekly HBPM Adherence feedback
-Table A7.1: Weekly HBPM adherence feedback SMSes (for Arm 2 participants)

Code	Frequency of HBPM	SMS Message Type	SMS Text Message
**B1**	≥3	Praise message	Dear participant, you monitored your BP at least 3 days this week. Congratulations, keep it up! A gentle reminder to upload your BP immediately after measurement. Please ensure that Internet (Wi-Fi/data) and Bluetooth is switched on. Thank you.
**B2**	2	Encouraging message	Dear participant, you monitored your BP 2 days this week. Measure your BP 3 times/week for better health. A gentle reminder to upload your BP immediately after measurement. Please ensure that Internet (Wi-Fi/data) and Bluetooth is switched on. Thank you.
**B3**	1	Encouraging message	Dear participant, you monitored your BP 1 day this week. Measure your BP 3 times/week for better health. A gentle reminder to upload your BP immediately after measurement. Please ensure that Internet (Wi-Fi/data) and Bluetooth is switched on. Thank you.
**B4**	0	Reminder message	Dear participant, you did not monitor your BP this week. Measure your BP 3 times/week for better health. Please call the CRC at XXXX XXXX if you experience any technical difficulties (Mon-Fri, 8:30am-4:30pm). A gentle reminder to upload your BP immediately after measurement. Please ensure that Internet (Wi-Fi/data) and Bluetooth is switched on. Thank you.

-Table A7.2: Weekly HBPM adherence feedback SMSes [for Arm 3a (Instant Reward) participants]

Code	Frequency of HBPM	SMS Message Type	SMS Text Message
**C1**	≥3	Praise message	Dear participant, you monitored your BP at least 3 days this week, earning $9. Total earned: $Y. Congratulations, keep it up! A gentle reminder to upload your BP immediately after measurement. Please ensure that Internet (Wi-Fi/data) and Bluetooth is switched on. Thank you.
**C2**	2	Encouraging message	Dear participant, you monitored your BP 2 days this week, earning $6. Total earned: $Y. Measure your BP 3 times/week for better health. A gentle reminder to upload your BP immediately after measurement. Please ensure that Internet (Wi-Fi/data) and Bluetooth is switched on. Thank you.
**C3**	1	Encouraging message	Dear participant, you monitored your BP 1 day this week, earning $3. Total earned: $Y. Measure your BP 3 times/week for better health. A gentle reminder to upload your BP immediately after measurement. Please ensure that Internet (Wi-Fi/data) and Bluetooth is switched on. Thank you.
**C4**	0	Reminder message	Dear participant, you did not monitor your BP this week, not earning any reward. Total earned: $Y. Measure your BP 3 times/week for better health. Please call the CRC at XXXX XXXX if you experience any technical difficulties (Mon-Fri, 8:30am-4:30pm). A gentle reminder to upload your BP immediately after measurement. Please ensure that Internet (Wi-Fi/data) and Bluetooth is switched on. Thank you.

-Table A7.3: Weekly HBPM adherence feedback SMSes [for Arm 3b (Health Capital) participants]

Code	Frequency of HBPM	SMS Message Type	SMS Text Message
**D1**	≥3	Praise message	Dear participant, you monitored your BP at least 3 days this week. Your capital increased by $X and now amounts to $Y. Congratulations, keep it up! A gentle reminder to upload your BP immediately after measurement. Please ensure that Internet (Wi-Fi/data) and Bluetooth is switched on. Thank you.
**D2**	2	Encouraging message	Dear participant, you monitored your BP 2 days this week. Your capital decreased by $X and now amounts to $Y. Measure your BP 3 times/week for better health. A gentle reminder to upload your BP immediately after measurement. Please ensure that Internet (Wi-Fi/data) and Bluetooth is switched on. Thank you.
**D3**	1	Encouraging message	Dear participant, you monitored your BP 1 day this week. Your capital decreased by $X and now amounts to $Y. Measure your BP3 times/week for better health. A gentle reminder to upload your BP immediately after measurement. Please ensure that Internet (Wi-Fi/data) and Bluetooth is switched on. Thank you.
**D4**	0	Reminder message	Dear participant, you did not monitor your BP this week. Your capital decreased by $X and now amounts to $Y. Measure your BP3 times/ week for better health. Please call the CRC at XXXX XXXX If you experience any technical difficulties (Mon-Fri, 8:30am-4:30pm). A gentle reminder to upload your BP immediately after measurement. Please ensure that Internet (Wi-Fi/data) and Bluetooth is switched on. Thank you.

-Table A7.4: Incentive scheme for Arm 3 participants

	**Group 3a: Instant Reward**	**Group 3b: Health Capital**
Starting amount	$0	$72
Days with BP measures in the week<br>3 or more days<br>2 days<br>1 day<br>0 day	 $ 9 earned<br>$ 6 earned<br>$ 3 earned<br>$ 0 earned	 ▲ $6 higher<br>▼ 10% lower<br>▼▼ 20% lower<br>▼▼▼ 30% lower
Maximum total earnings (after 24 weeks)	$216	$216

Note: Participants' Health Capital amount would be calculated and rounded off to the nearest dollar (e.g. $39.31 will be rounded off to $39).

-Figure A7.1. Weekly HBPM Adherence Feedback

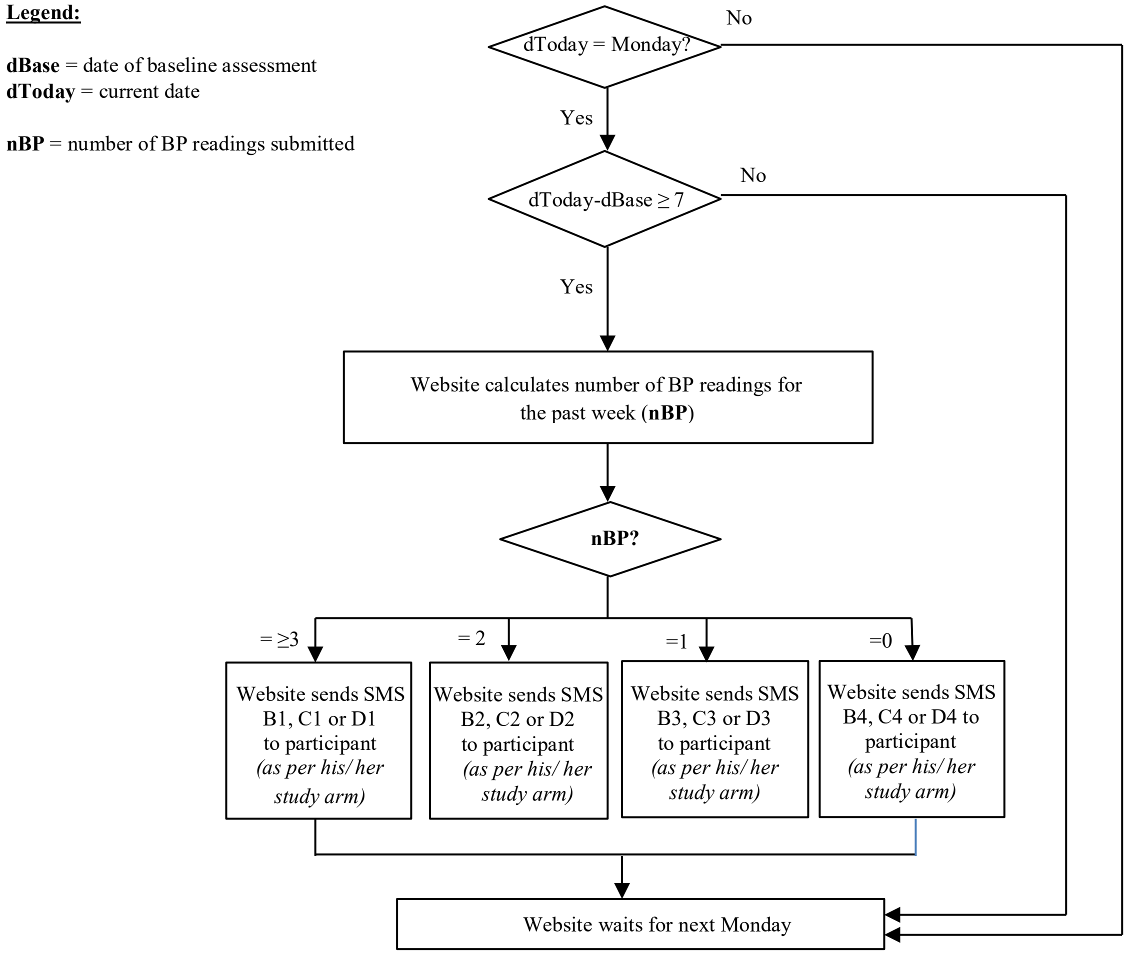

Supplement: Multimedia Appendix 7 [file resprot_v10i6e27496_app7.pdf]
