# Supplementary material for: Wireless Home Blood Pressure Monitoring System With Automatic Outcome-Based Feedback and Financial Incentives to Improve Blood Pressure in People With Hypertension: Protocol for a Randomized Controlled Trial
Source: JMIR Res Protoc. 2021 Jun 9;10(6):e27496. doi: 10.2196/27496 (PMC8262550; doi:10.2196/27496)
Supplement: Multimedia Appendix 8 [file resprot_v10i6e27496_app8.pdf]

Multimedia Appendix 8: 28-day continuous HBPM assessment

-Figure A8.1: Black Protocol (Clinical Protocol for Very Low- and Extremely High- Average BP)

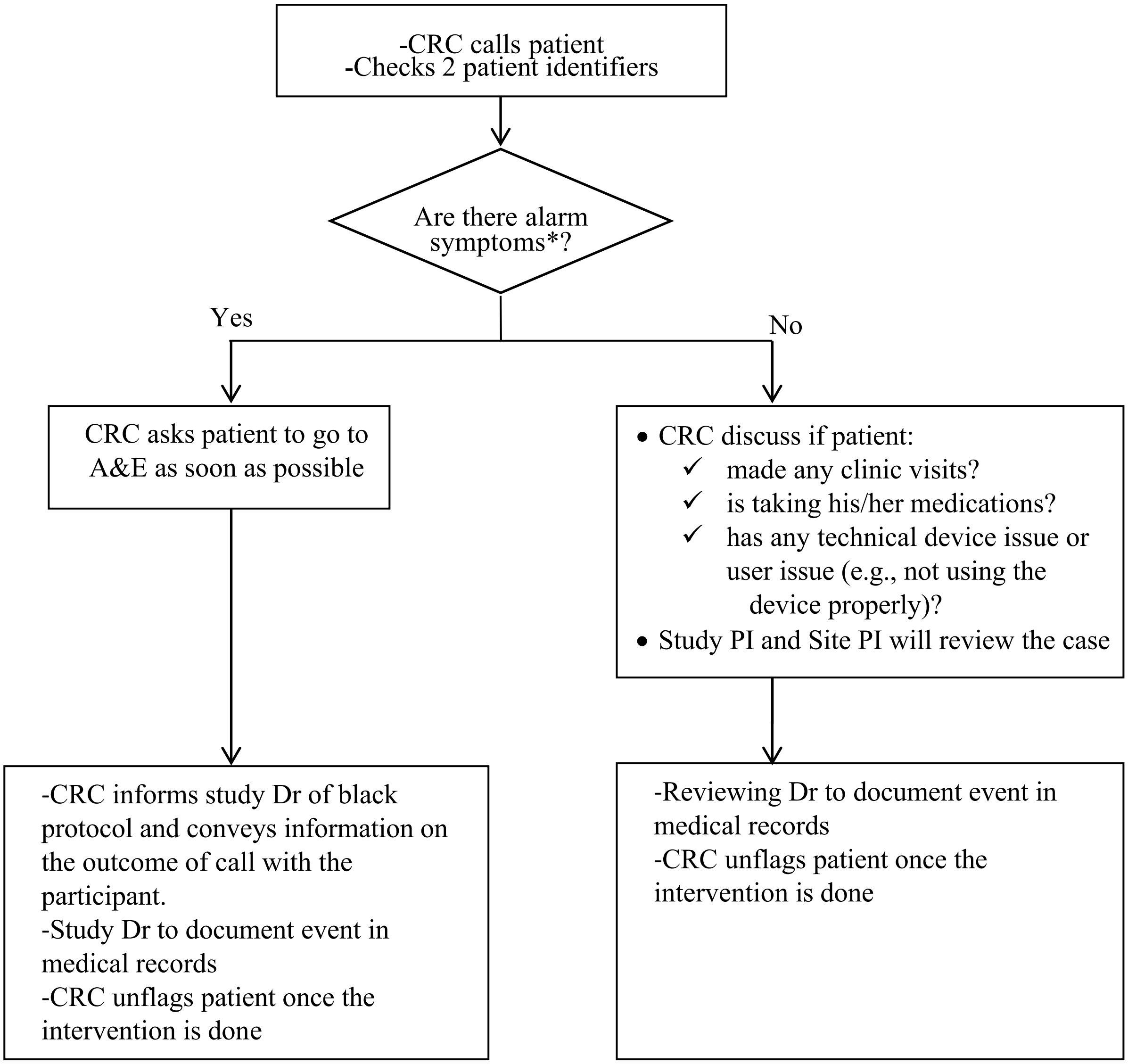

<u>**Legend**</u>:
A&E = Accident and Emergency
CRC = Clinical Research Coordinator
PI = Principal Investigator

*__Alarm symptoms__= postural dizziness, breathlessness, or chest pain

-Figure A8.2: Gray Protocol (Clinical Protocol for Low Normal Average BP)

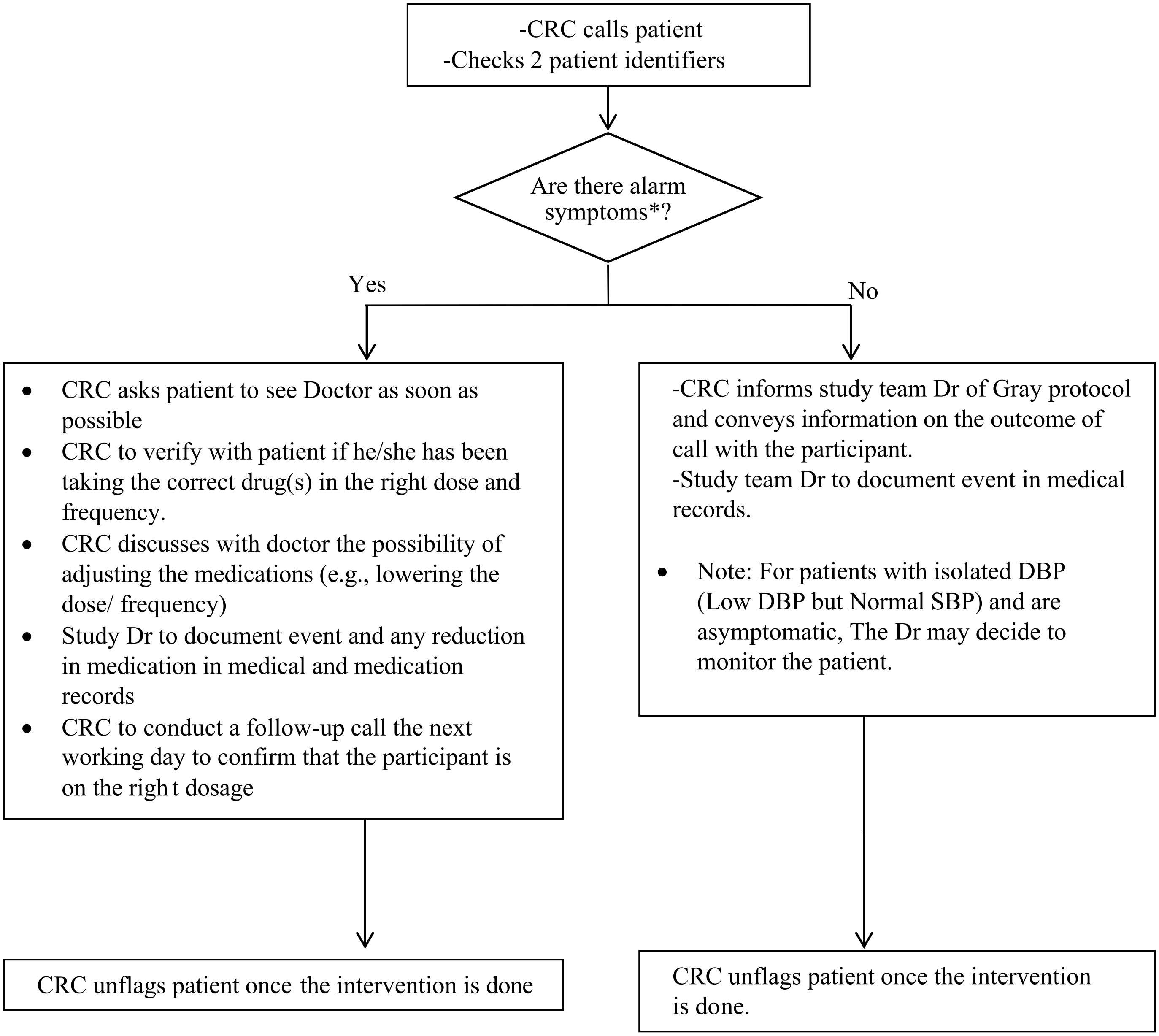

Legend:
CRC = Clinical Research Coordinator
***Alarm symptoms**= postural dizziness, breathlessness, or chest pain

-Figure A8.3: Green Protocol (Clinical Protocol for Normal Average BP)

CRC flags patient to the doctor as candidate for skipping upcoming clinic visit

-Doctor reviews the case (e.g. comorbidities, pending blood tests), and makes clinical judgment whether upcoming clinic visit will be skipped or not
-Reviewing Dr to document event in medical records

Yes = Upcoming clinic visit to be skipped

- CRC calls patient (Checks 2 patient identifiers)
- CRC checks with patient if he/she has any side effects, e.g. postural dizziness, breathlessness and chest pain.

- If No, CRC proposes cancellation of upcoming clinic appointment and proposes collection of medication via PILBOX (if patient did not opt out) or from the pharmacy.
- If patient is agreeable to cancellation, CRC will cancel patient's scheduled appointment.
- Study Dr to prescribe all medication that patient is currently on for the next 3 months or until the next appointment.
- Study Dr to document event in medical records.
- CRC to follow clinic's workflow for medication collection without Dr consultation.
- For collection via PILBOX: CRC to put prescription in pharmacy.
- For collection at Pharmacy: CRC to meet patient to pass prescription.
- The CRC will book an appointment for the participant's next visit to consult the Dr.
- CRC to conduct a follow-up call the next working day to confirm that patient has collected medicine and is taking the drug correctly.

- If Yes, CRC to advise patient to attend appointment.
- CRC informs st udy Dr on the outcome of call with the participant.
- -St udy team Dr to document event in medical records

-CRC unflags patient once the intervention is done

No = Upcoming clinic visit to be retained

- CRC calls patient (Checks 2 patient identifiers)
- CRC reminds patient of upcoming clinic visit

-CRC informs study Dr on the outcome of call with the participant.
-Study team Dr to document event in medical records
-CRC unflags patient once the intervention is done

<u>**Legend**</u>:
CRC = Clinical Research Coordinator
PILBOX = Prescription in a Locker Box

-Figure A8.4: Pink Protocol ((Clinical Protocol for Slightly High- and Very High- Average BP)

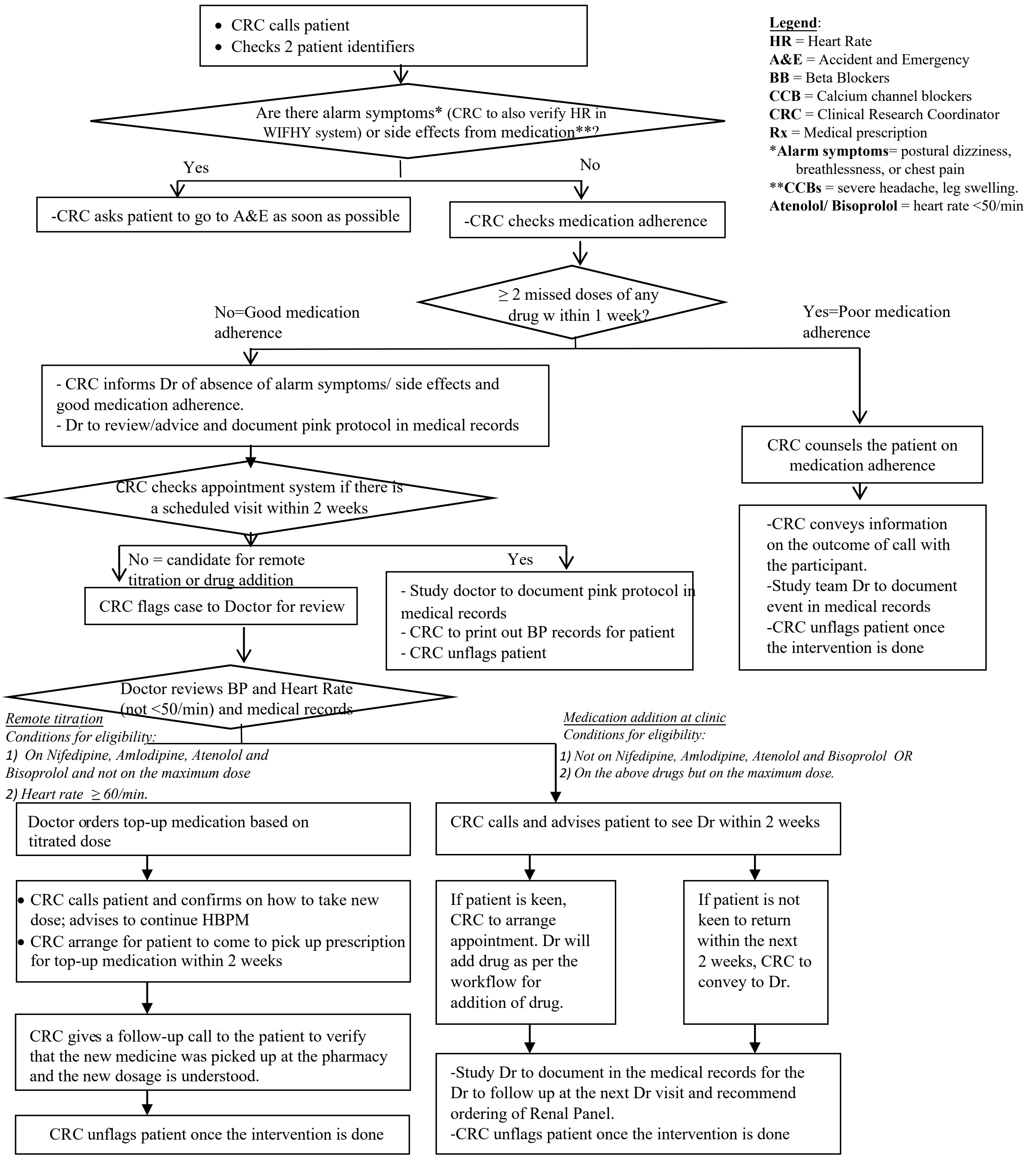

-Figure A8.4.1: Pink Protocol- Remote anti-hypertensive drug titration workflow

Doctor visit 1 (Baseline)

1. Doctor indicates in medical and medication records the anti-hypertensive[1] drug and dose for titration.
2. Refers to CRC

CRC visit (Baseline)

1. Tag drug to be titrated with orange sticker, reinforce to patient with PIL
2. Advise that dose/ frequency may be increased if BP not to target. Coach how to titrate drug.
3. Advice on follow-up plan with CRC

Day 0

Baseline

CRC call (titration)[2]
(4 week mean HBP 135 – 179/ 85[80]-109, [DM])

1. Check 2 patient identifiers
2. Check for alarm symptoms/ side effects with new medication[3]?
3. Check adherence to drug(s) (skip ≥2 dose per week?)
4. No BP < 90/50 mmHg in the last 4 weeks.
5. Mean Diastolic BP ≥ 60 mmHg
6. Titrate pre-determined drug, in a stepwise manner

i. If titrated up: Confirm how patient will take new dose. Continue HBPM. Doctor to update medication records about titration.
ii. Arrange for patient to come to pick up prescription for titrated medication within 2 weeks
iii. If Doctor decides to add new anti-hypertensive drug, arrange for patient to see Doctor in 2 weeks.

Top up of Rx at Pharmacy (2 weeks after call)

- Patient returns to collect top-up of the new anti-hypertensive drug(s).
- Pharmacy staff to confirm dosage with patient

4 weekly review of HBPM

Titration – CRC/Doctor

2 weeks after call

Patient tops up drug at Pharmacy

Doctor visit 2
1. Review HBPM
2. Note any remote dose titration → check how patient is taking the anti-hypertensive drug(s).
3. Prescribe anti-hypertensive drugs accordingly (e.g. add new drug or increase dose of old ones).

Weeks 12-24

Doctor Visit

Legend:
BB=Beta blockers; **CC**=Calcium channel blockers; **CRC**=Clinical Research Coordinator; **DM**=diabetes; **OM**= every morning; **ON**= every night; **PIL** = Remote Titration Plan-Patient Information Leaflet; **Rx** = Medical prescription;
1. Amlodipine ≤ 7.5 mg/day, Nifedipine LA ≤ 30mg/day, Atenolol ≤ 75 mg OM and Bisoprolol ≤ 7.5 mg OM at recruitment.
2. Refer to "Pink Protocol" of WIFHY Study;
3. Side effects: Postural dizziness, breathlessness, chest pain. CCBs: severe headache, leg swelling. Atenolol/ Bisoprolol: heart rate < 50/min

-Figure S4: Pink Protocol- Drug selection workflow for remote titration
(*Unless decided otherwise by study team Dr)

A) Look for a CCB 1st. If absent or at maximum dose, then look for BB.<br><br>B) Create a titration plan as per the protocol shown below.

1) Nifedipine LA

- 30 mg OM/ON --> 60 mg OM/ON or 30 mg BD

- 60 mg OM --> 60 mg OM and 30 mg ON

- 30 mg BD --> 60 mg OM and 30 mg ON

- 60 mg OM/ON and 30 mg ON/OM --> 60 mg BD

2) Amlodipine

- 2.5 mg OM/ON --> 5 mg OM/ON

- 5 mg OM/ON --> 7.5 mg OM/ON or 5 mg OM + 2.5 mg ON

- 7.5 mg OM/ON --> 10 mg OM/ON or 5 mg BD

3) Atenolol (Note: Average pulse should be ≥ 60/min)

- 25 mg OM/ON --> 50 mg OM/ON or 25 mg BD

- 50 mg OM/ON --> 75 mg OM/ON or 50 mg OM + 25 mg ON

- 75 mg OM/ON--> 100 mg OM/ON or 50 mg BD

4) Bisoprolol (Note: Average pulse should be ≥ 60/min)

- 2.5 mg OM/ON --> 5 mg OM/ON

- 5 mg OM/ON --> 7.5 mg OM/ON or 5 mg OM + 2.5 mg ON

- 7.5 mg OM/ON --> 10 mg OM/ON or 5 mg BD

-Figure A8.4.1.2: Pink Protocol- Remote Titration Action Plan Patient Information Leaflet (PIL)

Wireless monitoring and financial incentives for uncontrolled hypertension (WIFHY)

Remote Titration Action Plan Patient Information Leaflet (PIL)

Your Blood Pressure (BP) readings were monitored remotely. As your BP was persistently high, your Dr has changed your medication dose. You are currently taking the medicine in the GREEN zone.

As you have been advised to change your medication dose/ frequency, please follow the dose in the YELLOW zone.
Sometimes, if you have to take 2 tablets of your old drug, you may be given a bigger tablet. This may be of a different colour from your old drug.

**Medication** (please tick)	**You are NOW TAKING:**		**Medication** (please tick)	**As advised by the Dr, please CHANGE to:**	
	**Morning**	**Evening**		**Morning**	**Evening**
☐ **Nifedipine LA 30mg**	___ **mg**	___ **mg**	☐ **Nifedipine LA 30mg**	___ **mg**	___ **mg**
☐ **Nifedipine LA 60mg**	___ **mg**	___ **mg**	☐ **Nifedipine LA 60mg**	___ **mg**	___ **mg**
☐ **Amlodipine 5mg**	___ **mg**	___ **mg**	☐ **Amlodipine 5mg**	___ **mg**	___ **mg**
☐ **Amlodipine 10mg**	___ **mg**	___ **mg**	☐ **Amlodipine 10mg**	___ **mg**	___ **mg**
☐ **Atenolol 50mg**	___ **mg**	___ **mg**	☐ **Atenolol 50mg**	___ **mg**	___ **mg**
☐ **Atenolol 100mg**	___ **mg**	___ **mg**	☐ **Atenolol 100mg**	___ **mg**	___ **mg**
☐ **Bisoprolol 2.5mg**	___ **mg**	___ **mg**	☐ **Bisoprolol 2.5mg**	___ **mg**	___ **mg**
☐ **Bisoprolol 5mg**	___ **mg**	___ **mg**	☐ **Bisoprolol 5mg**	___ **mg**	___ **mg**

-Figure A8.4.2: Workflow for addition of other classes of anti-hypertensives
[(i.e. Angiotensin Converting Enzyme Inhibitor (ACEIs), Angiotensin Receptor Blockers (ARBs) and Diuretics] at Doctor visit

[Notes: 1) Is applicable for participants who are ineligible for Remote Anti-hypertensive Drug Titration
2) Please kindly note that addition of drugs is not to be carried out remotely. It is to be carried out when reviewing the patient in the clinic.
3) Please note that this workflow is only applicable for the pink protocol which involves the addition of other classes of Anti-hypertensives (i.e. ACEIs, ARBs and Diuretics). The workflow will not be activated if the addition of anti-hypertensives (i.e. ACEIs, ARBs and Diuretics) is done as part of clinical care.]

For Addition of ACEI/ ARBs:
- To have Renal Panel done within 2-4 weeks of drug initiation
- For normal Renal Panel results, for patient to be informed of results via phone call
- For abnormal Renal Panel results [i.e. Creatinine (Cr), Potassium (K)], for patient to come in person to see a Doctor.

For Addition of Diuretics:
- To have Renal Panel done within 12 weeks of drug initiation.
- For normal Renal Panel results, for patient to be informed of results via phone call.
- For abnormal Renal Panel results [i.e. Sodium (Na), Potassium (K) and Chloride (Cl)], for patient to come in person to see a Doctor.

-Figure A8.5: Yellow Protocol (Clinical Protocol for zero BP readings detected in the WIFHY system)

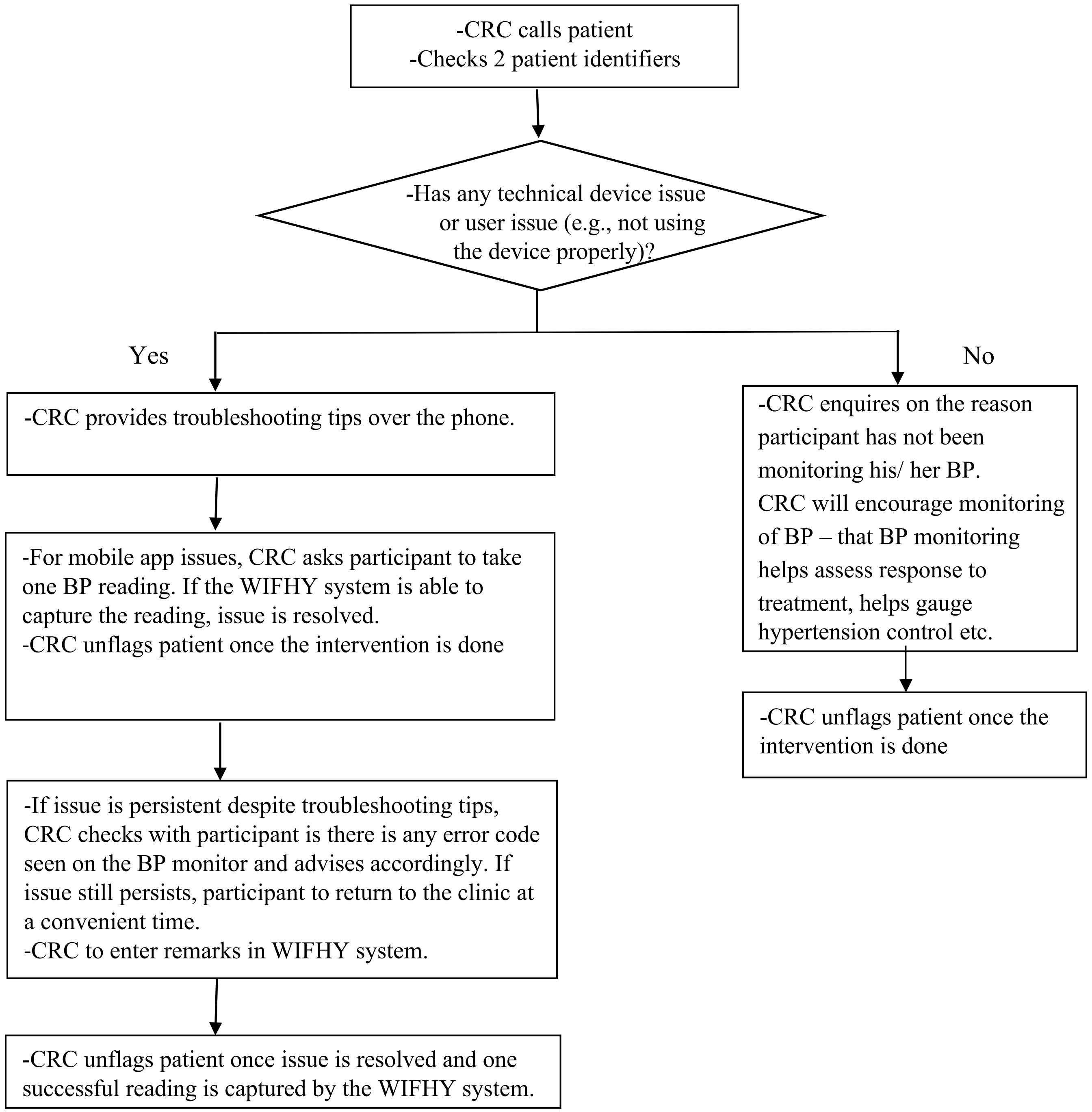

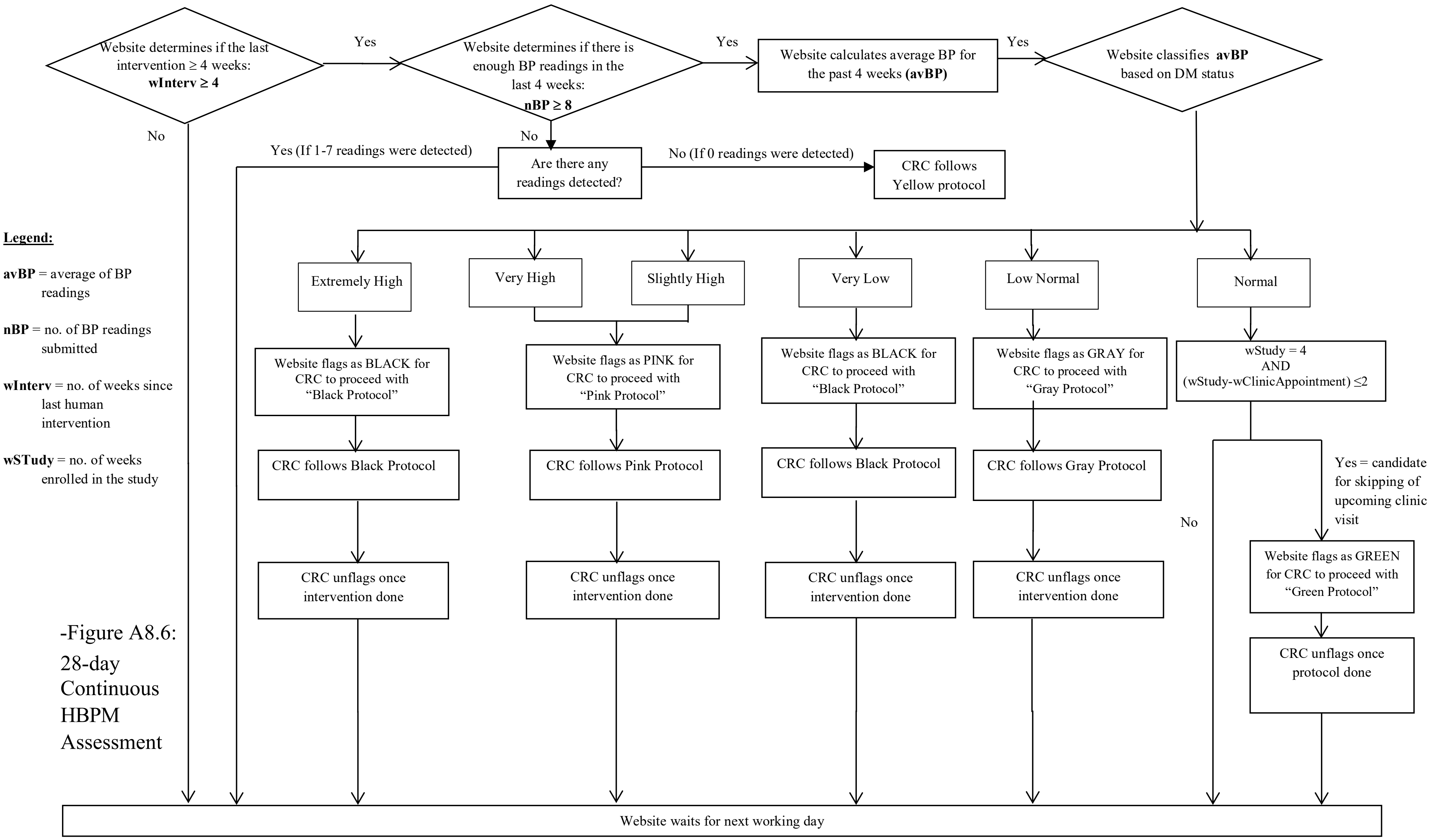

Legend:

avBP = average of BP readings

nBP = no. of BP readings submitted

wInterv = no. of weeks since last human intervention

wSTudy = no. of weeks enrolled in the study

-Figure A8.6: 28-day Continuous HBPM Assessment
