# Supplementary material for: Wireless Home Blood Pressure Monitoring System With Automatic Outcome-Based Feedback and Financial Incentives to Improve Blood Pressure in People With Hypertension: Protocol for a Randomized Controlled Trial
Source: JMIR Res Protoc. 2021 Jun 9;10(6):e27496. doi: 10.2196/27496 (PMC8262550; doi:10.2196/27496)
Supplement: Multimedia Appendix 9 [file resprot_v10i6e27496_app9.pdf]

Multimedia Appendix 9: Study payment scheme

-Table A9.1: Study payment scheme for participants

	**Arm 1**	**Arm 2**	**Arm 3**
Completion of baseline assessment	$25	$25	$25
Attending all study visits	$25	$25	$25
Fairness payment	$100	$100	-
Adherence to blood pressure monitoring	-	-	Varies (up to $216)
Telecommunication costs	-	$30	$30
