# Supplementary material for: Wireless Home Blood Pressure Monitoring System With Automatic Outcome-Based Feedback and Financial Incentives to Improve Blood Pressure in People With Hypertension: Protocol for a Randomized Controlled Trial
Source: JMIR Res Protoc. 2021 Jun 9;10(6):e27496. doi: 10.2196/27496 (PMC8262550; doi:10.2196/27496)
Supplement: Multimedia Appendix 10 [file resprot_v10i6e27496_app10.pdf]

Multimedia Appendix 10: Outcome measures

-Table A10.1: Outcome measures and schedule of collection

**Measurement/survey instrument**	**Construct measured**	**Baseline**	**Month 6**
EQ-5D-5L (EuroQol Group)	Quality of life	√	√
Brief Illness Perception Questionnaire-Revised (BIPQ-r)	Perceptions of hypertension	√	√
Global Physical Activity Questionnaire (GPAQ)	Physical activity and sedentary behavior	√	√
Dietary Practices Questionnaire (DPQ)	Dietary practices	√	√
Healthcare services expenditure	Utilization of healthcare services	√	√
Treatment satisfaction questionnaire for HBPM	Treatment satisfaction for home BP monitoring (wireless and non-wireless systems)		√
Socio-demographics		√	
