# Supplementary material for: Wireless Home Blood Pressure Monitoring System With Automatic Outcome-Based Feedback and Financial Incentives to Improve Blood Pressure in People With Hypertension: Protocol for a Randomized Controlled Trial
Source: JMIR Res Protoc. 2021 Jun 9;10(6):e27496. doi: 10.2196/27496 (PMC8262550; doi:10.2196/27496)
Supplement: Multimedia Appendix 11 [file resprot_v10i6e27496_app11.pdf]

Proposal S/N: HSRG13MAY009

Principal investigator: Dr Marcel Bilger **Institution:** Duke-NUS

Proposal title: **Randomized control trial to improve hypertension outcomes using wireless home blood pressure monitoring with automatic outcome-based feedback and financial incentives**

Please respond to reviewer comments as far as possible, making use of the box to the right below.

Reviewer Comments	PI’s Response
**Literature Review**<br>1. Suggest reviewing the results of AL Kinmonths’ work in the UK wherein she conducted a trial of complex behavioural intervention for prevention of diabetes which failed to show benefit.	1. Kinmonth et al (1) investigated whether two facilitated theory-based behavioural interventions (telephone- and home-based) were more effective than a brief advice leaflet at increasing physical activity. The sample included 365 sedentary adults who had a parental history of diabetes and both interventions lasted 1 year. The telephone-based intervention included four 45-min calls and two 15-min support calls during the 5-months' intensive phase, followed by monthly postal contact for the remaining 7 months. The home-based intervention included four 1-hour home visits and two 15-min telephone calls during the 5-month intensive phase, and monthly 30-min follow-up phone calls for the rest of the year. The authors found that at 1 year, the physical-activity ratio of participants who received the intervention, in either delivery mode, did not differ from the ratio of those who were given a brief advice leaflet.

Reviewer Comments	PI's Response
**Feasibility**	Our proposed multi-faceted intervention is quite different from the Kinmonth et al. study. as it targets a different health condition (hypertension vs. diabetes) and a different behaviour (blood pressure monitoring and management vs. physical activity). More importantly, it applies a different and more comprehensive strategy that aims at levering the full potential of wireless home pressure monitoring (wireless transmission of results, automatic reminders, advice and reinforcement, flagging uncontrolled or incompliant patients and remote titration, and financial incentives).<br>We think that our proposed intervention is likely to be effective as blood pressure monitoring has been shown to successfully reduce blood pressure (2-4), even among non-adherent patients (5). Our interventions aim to increase adherence to home blood pressure monitoring, and thereby health outcomes, by combining self-monitoring with instant outcome-based reinforcement and incentives.

Reviewer Comments	PI's Response
2. Lack of demonstrations of study feasibility:<br>a. There is no indication of patient involvement in design of a project that requires sustained effort from participants.<br>b. Has there been a pilot study of feasibility?<br>c. Participation requires daily performance of BP monitoring, and paper recording by individuals in the non-device arm. Is there any evidence that patients will sustain their involvement?<br>d. Is it feasible to recruit 228 patients with uncontrolled hypertension in the clinic over 18 months? To include the prevalence of patients who meet the study criteria and a timeline for recruitment.	2a. Patient involvement is essentially the same in the study compared to usual care at the SingHealth polyclinic in Bedok where patients are instructed to self-monitor their BP 3 times a week and record the results manually. It is true that self-monitoring requires patient involvement, which is precisely what the study is trying to improve using the comprehensive strategy described in our response to Comment 1. In particular, patients in interventions arms will benefit from greater support to sustain their self-monitoring through BMP-related feedback (Arms 2&3) and financial incentives (Arm 3).<br>2b. Dr Juliana Bahadin, the Site PI, was part of a team that conducted a pilot trial on chronic disease management through telecare that notably involved hypertensive patients from one SingHealth polyclinic. It was found that participation rate was encouraging (57 of 82 eligible patients participated) and patient involvement satisfactory (6).<br>In addition, the feedback loop between the patient's wireless HBPM and the clinic using MOHH's National Health Platform (NHP) architecture is now operational for iPhone users and a solution for Android-supported phones is expected to be available in 2015.<br>2c. Please refer to our response to Comment 2a concerning patient involvement in the intervention. A related issue is patient involvement in the study as it is possible that some participants in the control arm will monitor their blood pressure but omit to record the results on paper. As pointed out in our response to Reviewer Comment 2a, paper recording is part of usual care, and lack thereof is part of the non-adherence behaviour which the interventions aim at reducing. Note that in the proposed study, it will be possible to distinguish between non-adherence to blood pressure monitoring and non-adherence to paper recording in most of the cases as the digital home blood pressure monitors can store the last 30 readings (10 weeks worth of monitoring at a rate of 3 times a week).<br>Finally, to further improve patient involvement in the study and promote retention across all study arms, we propose to add a $10 monetary reward for completing the baseline questionnaire, and increase the monetary reward at follow-up from $30 to $50. See point 27 below for our proposed budget amendments.<br>2d. We plan recruiting 228 participants in 12 months. The SingHealth polyclinic in Bedok looks after 40,000 hypertensive patients, 40% of which have [illegible]

Reviewer Comments	PI's Response
3. It will be important to monitor how much additional work is generated for the nurses from the polyclinic and how much time it takes to provide the necessary clinical assessment and feedback, in order for this trial to have transferability to clinical settings without research funding.	3. We fully agree with the Reviewer's comment. Nurse and Doctor time per patient will be monitored in each study group as part of our Cost-Effectiveness Analysis (as describe in the secondary outcomes section of the grant proposal). Note that the intervention involves skipping the Month 3 visit for well-controlled patients and thus could potentially lead to time and cost savings. Analysing such costs is one of the secondary aims of the proposed study.
**Study design**	

Reviewer Comments	PI's Response
4. The reviewer has main concern about the length of intervention and lack of follow-up:<br>a. Will the outcomes persist after the intervention is stopped?<br>b. Have the investigators considered that financial incentives are needed for life or only for the study period?<br>c. If a difference is found, will it recede without the intense support or financial rewards?	4a. The intervention is not meant to test habit formation but to test the effect of sustained use of a Wireless home blood pressure monitor system, and of such system with additional financial incentives. From that perspective, the study design needs no follow-up assessment.<br>4b. If our study were to show that financial incentives are cost-effective, yes implementing the intervention in real life would amount to providing life-time financial incentives. While this is ultimately a political decision, a sustainable solution would be to draw the financial resources from the subsidies that are already in place, and to make part of these subsidies conditional on treatment adherence. This would not only have the advantage of nudging patients towards greater treatment adherence and better health outcomes but also of making a more cost-effective use of public subsidies by focusing them on those treatments that are being effectively used.<br>4c. For the above reasons, the proposed study is not designed to measure post-intervention effectiveness. That said, the study team is open to add a month 12 assessment to the study, that is 6 months after discontinuation of the financial incentives to measure habit formation. However, a consequence is that it would make the study last more than the maximum of two years (12 months recruitment + 6 months financial incentives + 6 months without financial incentives + 6 months for data analysis, checks and write-up = 30 months) and increase labour costs by 97.5k. The study team would be very pleased to discuss potential solutions if MOH were interested in the measurement of habit formation through financial incentives.

Reviewer Comments	PI's Response
5. How will random recruitment occur?	5. The recruitment process will be finalized when writing the study protocol. At this point in time it is expected to take place as follows:<br>Participants will be randomly allocated to 1 of 3 study arms of size 76 each. Following, Reviewer Comment 7c, we will stratify randomization according to patient risk category (high or low). Randomization will be performed separately for both risk categories, with block size of 9 patients to allocate the risk categories across all three arms as equally as possible. Randomization will be computer-based using the online generator Sealed Envelope™ and performed by two NUS team members. The two NUS team members will then create two sets of participant unique ID numbers, one per risk category. They will label the envelopes with these ID numbers and risk category, and inside each envelope, they will place a slip of paper with the randomly-generated arm allocation for that ID number. The NUS team members will then seal the envelopes and pass them to the Research Nurse at the Bedok Polyclinic.<br>At the polyclinic, doctors will refer interested patients with uncontrolled blood pressure to the Research Nurse who will verify the remaining eligibility criteria and take informed consent. The Research Nurse will then assign each successfully enrolled participant a unique ID number corresponding to the patient's risk category. Based on the participants unique ID number the nurse will select an envelope with the corresponding ID. The envelope will contain a sheet of paper with the participant's group allocation.

Reviewer Comments	PI's Response
6. Concerns on patients selection:<br>a. By prioritising patients who own i-phones to wireless monitoring arms, will that not constitute a bias towards wealthier and more educated patients?<br>b. It is unclear how they will select patients who require loaner phones (i.e. those who do not own i-phones) and train them to use the devices.	6a. We do not propose prioritizing patients who own i-phones to wireless monitoring arms as this would bias the comparison with usual care. What we propose is to include having a compatible smartphone as an inclusion criteria that applies to all study arms. Currently only IPhones are supported but Android-based smartphones are expected to be supported starting 2015, which will accelerate recruitment by increasing the pool of eligible patients.<br>6b. Using loaner phones is a contingency plan in case patient recruitment was more challenging than anticipated. If this contingency plan were used, all patients fulfilling all other eligibility criteria will be asked to participate in the study using a loaner phone. It is true that such patients will require more training by the Research Nurse. This is not our preferred option and we will do our best to recruit all patients according to point 6a.
7. [Page 5] Experimental design:<br>a. Clarify if the study will only recruit newly diagnosed patients or existing patients who meet the inclusion criteria.<br>b. Provide details on recruitment and randomisation processes.<br>c. Will there be any differences in the clinical outcomes between high and low risk patients? If so, the team should consider stratify the patients based on their risk before randomisation.	7a. Both new and existing patients will be eligible.<br>7b. See our response to Comment 5.<br>7c. Many thanks for this excellent suggestion. We will stratify randomization by risk group as this factor is very likely to influence systolic blood pressure at 6 month, which is the primary outcome of the proposed study.

Reviewer Comments	PI's Response
8. Lack of information on intervention:<br>a. There is no evidence of patient engagement strategies beyond what appears to be a single counselling session with a nurse educator.<br>b. What information will the trusted individuals receive? How will they be engaged in the process? Will they be present at the patient education visit?	8a. Compared to usual care, patients in the intervention arms will benefit from remote engagement during the whole period between doctor visits. The intensity and nature of engagement will depend on blood pressure outcomes. Engagement essentially consists in sending reinforcement messages to well-controlled patients, while warning messages, contacting a trusted person, phone calls from the Nurse, titration change, and additional doctor visits are available options for uncontrolled patients depending on their blood pressure readings.<br>8b. The trusted person will be contacted by the Project Coordinator and the study will be explained to her over the phone. The trusted person will be automatically informed by the system in case of lack of self-monitoring or severely out-of-range blood pressure readings. The role of the trusted person is to champion adherence and check on the patient in case of excessively low or high blood pressure readings. The nature and intensity of involvement is left at the discretion of the trusted person as it would if the intervention were to be scaled up.

Reviewer Comments	PI's Response
9. [Page 9] Sample size:<br>a. Provide reference on the sample size calculation method.<br>b. Justify the effect size of 10 mmHg in average SBP at 6 months.<br>c. Clarify if the team assumed the same effect size (i.e. 10 mmHg) will be observed between Arm 1 and Arm 2, and between Arm 2 and Arm 3. Otherwise, separate sample size calculations will be required and the final sample size should be selected based on the smallest effect size.<br>d. Does the sample size of 76 per arm include the buffer for the 20% attrition?	9a. The calculation is similar to the 2-sample t-test with the only difference being the use of a significance level alpha of 0.05/3 = 0.016 to account for 3 arms. Reference: Sample Size Tables for Clinical Studies, David Machin, Michael Campbell, Say Beng Tan and Sze Huey Tan, Wiley-Blackwell, Third Edition, 2008.<br>9b. The Framingham risk score predicts that for a female age 60 with high cholesterol, a reduction of systolic blood pressure of 10 mmHg from above normal levels (i.e. greater than 140 mmHg) reduces risk of a cardiovascular disease by around 1.5%. (7) Variations in risk reduction generated by reducing blood pressure depend on age, cholesterol level, gender, smoking status and whether the patient is on hypertensive medication. However it is clear that even a 10 mmHg reduction in systolic blood pressure has the potential to reduce risk of cardiovascular disease and events.<br>9c. The assumption is that the effect size is the same.<br>9d. Yes, the sample size of 76 includes the buffer for attrition.
10. [Page 6 & 7] Clarify what is a "test day"?	10. A test day is a day when a blood pressure measurement is scheduled to take place.
11. [Page 7] Outcomes:<br>a. Primary outcome of SBP have not defined clearly. Specify if the primary outcome is referring to "the change in SBP at 6-month from baseline". Similar comments for DBP.<br>b. Framingham risk score is well-validated tool for men and women aged 30-79. Will this tool be used for patient aged 20-29 years old?	11a. The primary outcome is SBP at month 6. To clarify we propose amending the study hypotheses as follows:<br>**H1**: The average systolic blood pressure at month 6 will be lower for the patients in the Wireless HBPM arm compared to the UC patients.<br>**H2**: The average systolic blood pressure at month 6 will be lower for the patients in the Wireless HBPM with Incentives arm compared to those in the Wireless HBPM are.<br>11b. We have decided not to use the Framingham risk score (see our response to Reviewer Comment 13).

Reviewer Comments	PI's Response
12. [Page 8 Control variables] In page 2, the author states that international guidelines recommend lifestyle modifications (including maintaining a healthy body weight, adopting a healthy diet regular, moderate exercise and reducing alcohol intake) and lipid lowering therapy for high-risk individuals. However, exercise and alcohol intake have not been included as control variables.	12. We thank the Reviewer for pointing that out. We will record physical activity levels (in average daily MET minutes) and alcohol consumption (in average daily grams) in the baseline questionnaire.
**<u>Measurements</u>**<br><br>13. Not clear how the Framingham assessment will assist with outcomes if only blood pressure is being addressed.	13. It is true that since age is the main contributing factor to the Framingham score, such score will not be very sensitive to the intervention effect. Considering that the study includes plenty of other secondary outcomes, we propose not to monitor the Framingham score and focus on the other outcomes.
14. Baseline data does not include smoking which is a strong risk factor for CHD. It would be important to include this in comparing groups.	14. Many thanks for this excellent suggestion. We will collect smoking status and use this factor as a control variable in the main mixed model. We will also conduct explanatory analysis to determine how this factor is linked to adherence behaviours and blood pressure at month 6.
15. Items in the patient satisfaction survey are not included. Research has shown that patients prefer to see their health care provider regularly. It may be important to compare those that are advised they do not need to see their practitioner with those that are asked to return.	15. The patient satisfaction survey will include a selection of validated scales developed by Ware et al. (8) to measure patient satisfaction with care processes, as well as additional questions on whether patients would recommend the intervention to a relative, and whether they would continue in the intervention, if available (9). We thank the Reviewer for his/her suggestion and will add a comparative analysis of patient satisfaction and other outcomes for the two groups.

Reviewer Comments	PI's Response
16. The measurement of BP appears to include only two measurements. How will white coat hypertension be addressed? Does the BP device take an average of readings or just one? What method will be used to measure BP in the clinic?	16. It is true that measuring BP in the clinic is prone to white coat hypertension. To address this important comment, we propose to follow a recent study published in the BMJ (10) and use the device BpTRU which is a fully automated sphygmomanometer that records blood pressure by the oscillometric method. The device is designed to take an initial reading to verify that the cuff is properly positioned to obtain valid readings. The observer then leaves the patient alone, and five more readings are taken automatically at pre-specified intervals (we propose 1 minute intervals for our study as these have been shown to yield the same results as 2 minute intervals). Note that a rest period is not needed before the first reading. We propose measuring our primary outcome as the average of the 5 readings. The BMJ study has shown that such measure is significantly less affected by white coat hypertension and that the accuracy of this measure in relation to the awake ambulatory blood pressure was also significantly better when compared with manual office blood pressure.
17. It does not address the adverse effects of close monitoring of BP such as anxiety generated by close attention to measurement.	17. We expect anxiety to significantly diminish over time as patients get accustomed to monitoring. While baseline SBP measures will likely be affected by anxiety among new patients, Month 6 SBP measure, our primary outcome, should be considerably more robust to anxiety.
18. Suggest to make contingency to use paper in case the proposed changes are not in place at the time of the study	18. Unfortunately, we do not understand what the "proposed changes" are referring to. That said, we thank the Reviewer for highlighting the importance of contingency plans which we will keep in mind when developing the study protocol.
19. The participants will be required to buy the digital monitor.<br>a. Will this be a deterrent to joining the study? Will this be measured?<br>b. What is the actual cost to the patient for the OMRON Home BP Monitor? What if a patient cannot pay for the monitor? Will they be excluded?	19a. In the light of this comment and other comments made elsewhere, we have decided to fully subsidize all blood pressure monitors in this study (i.e. digital and wireless). Our proposed budget amendments are described in point 27 below.<br>19b. See our response to Comment 19a.

Reviewer Comments	PI's Response
**Analysis**<br><br>20. Is there intention to analyse male vs female outcomes and to stratify by age groups, since age and gender relate to adherence? If so, is there sufficient power in the proposed sample size?<br><br>21. [Page 8 Analysis] Provide details on analysis of healthcare cost.	20. It is true that patient characteristics are likely to influence both patient outcomes and adherence behaviours. Note that the study's primary aim is not to establish the interventions' effectiveness on patient subgroups and has not been powered for this. However, we can partly address the Reviewer's comment by conducting an explanatory analysis of the effect of gender, age and other relevant patient characteristics on treatment adherence and systolic blood pressure at month 6 using regression analysis.<br>21. Time spent by Doctors and Nurses on each patient will be recorded along the study and costs determined using an average wage. Time of non-medical staff (e.g. Project Coordinator) on activities related to providing the intervention will also be recorded and a cost calculated. Other factors are the cost differential between digital and wireless HBPM and patient-specific and fixed costs associated to the feedback loop system. Costs will be analysed both from the clinic perspective and from the patient's.
**Team**<br><br>22. A behavioural psychologist would be a useful addition, given the emphasis on adherence to monitoring, medication, exercise and nutritional recommendations.	22. We thank the Reviewer for the suggestion and propose adding Asst Prof Janet Schwarz (Tulane University) to the study team as a Consultant. Asst Prof Schwarz is a leading international experimental cognitive psychologist doing research on how to use insights from psychology and economics to encourage individuals to engage in healthy behaviors and ultimately reduce chronic disease. In particular, Asst Prof Schwarz focuses on improving health behaviors such as enhanced medication adherence, better nutrition, smoking cessation and increased exercise. (see biosketch and support letter in Appendix).
**Budget**	

Reviewer Comments	PI's Response
23. Not clear why the large budget is needed for travel between sites?	23. Transport is required to facilitate collaboration between the Duke-NUS and Geylang Polyclinic. The Duke-NUS Project Coordinator will typically have a weekly trip to the polyclinic to collect de-identified study material so that it can be treated and analyzed at Duke-NUS. Additional unregular trips will required, especially during the protocol development phase and at key milestones to perform quality checks. Also, the Duke-NUS Project Coordinator will provide additional help to the Geylang staff on a need basis.<br>However, we acknowledge that we might have over-provisioned for transportation and have re-adjusted the budget to accommodate 1.5 taxi rides each way per week on average.
24. Why are smart phones and data plans supported but not the monitors?	24. In the light of this comment and other comments made elsewhere, we have decided to fully subsidize all blood pressure monitors in this study (i.e. digital and wireless). Our proposed budget amendments are described in point 27 below.

Reviewer Comments	PI's Response
25. Budget for manpower:<br>a. Justify the engagement of Research Assistant/Associate (IT specialization) for 18 months since the person is only required to build the system to send automated text messages.<br>b. Justify the requirement for two full time staff (i.e. Research Assistant/Associate and research nurse) since there appears to be an overlap in their job scopes:<br>i. One research coordinator will be contracted to oversee the project on Singhealth side and coordinate with DukeNUS and Singhealth IT.<br>ii. Research Assistant/Associate will oversee the project and to assist the site coordinator to input data, recruitment and follow up of patients in addition to data analysis and manuscript writing	25a. We would like to clarify the role and activity rate of our proposed research staff:<br>**Research Associate at Duke-NUS (2 years at 100% FTE)**<br>At the beginning of the project, the Research Associate will prepare the Project Agreement between Duke-NUS and the Bedok Polyclinic, CIRB documents and study protocol as well as all preparer all the screeners, questionnaires and other study material required. Throughout the study, the Research Associate will have a weekly trip to the Bedok Polyclinic to collect research material, coordinate research and perform quality checks. The Research Associate will also monitor recruitment and analyse attrition and preliminary data. The Research Associate will also perform the final data analysis and draft all study reports and scientific manuscripts.<br><br>**Research Nurse at Bedok Polyclinic (18 months at 100% FTE)**<br><br>The Research Nurse will administer the baseline and follow-up assessments and in particular measure the patients' systolic blood pressure using the bpTRU device, which is the primary outcome of the study. She will also explain the intervention to the patients, show the participants how to use the home-based blood pressure monitor and how to interpret the results. The Research Nurse will also show how to use the smartphone application for the wireless blood pressure monitor, provide education materials to all patients on reducing blood pressure, demonstrate the use of electronic containers eCAPs and scan them to verify medication adherence.<br><br>**Project Coordinator at Bedok Polyclinic (18 months at 100% FTE)**<br><br>The Project Coordinator will oversee the recruitment effort and actively recruit herself, provide participants with information on the study and schedule all study-related appointments between the Research Nurse and the participants. She will assist the Duke-NUS Research Associate with all matters concerning the Project Agreement between Duke-NUS and the Polyclinic, CIRB documents and study protocol. She will also assist the Research Nurse with the baseline and

Reviewer Comments	PI's Response
26. Justify why 3 return cab rides to Bedok polyclinic per week for 18 months will be required.	26. See our response to Comment 23.<br>27. **Proposed budget amendments (all amounts are in SGD)**<br>- Wireless blood pressure monitors: 152 units now fully paid for the participants in study arm 2 & 3 at $270 each = $41,040 (budget increased by $10,640).<br>- Digital blood pressure monitors: Now 76 units fully paid for the participants in study arm 1 at $70 each = $5,320 (new budget line).<br>- Automated sphygmomanometer bpTRU BPM-200: 1 unit at estimated $1,800 = $1,800 (new budget line).<br>- Hiring of a behavioral psychologist as a Consultant: $2,400 (new budget line).<br>- Transportation between Duke-NUS and Bedok Polyclinic: $3,510 (budget reduced by $3,510).<br>- Monetary reward to patients who complete the baseline questionnaire: 228 patients times $10 = $2,280 (new budget line).<br>- Incentive to attend follow-up assessment: 228 patients times $50 = $11,400 (budget increased by $4,560).<br>- All other budget lines remain unchanged.<br>- All proposed changes increase the budget by $23,490, for a total budget of **$513,775**.

1. Kinmonth A-L, Wareham NJ, Hardeman W, Sutton S, Prevost AT, Fanshawe T, et al. Efficacy of a theory-based behavioural intervention to increase physical activity in an at-risk group in primary care (ProActive UK): a randomised trial. The Lancet.371(9606):41-8.
2. Ogedegbe G, Schoenthaler A. A systematic review of the effects of home blood pressure monitoring on medication adherence. The Journal of Clinical Hypertension. 2006;8(3):174-80.
3. Bray EP, Holder R, Mant J, McManus RJ. Does self-monitoring reduce blood pressure? Meta-analysis with meta-regression of randomized controlled trials. Annals of medicine. 2010;42(5):371-86.

4. Fahey T, Schroeder K, Ebrahim S, Glynn L. Interventions used to improve control of blood pressure in patients with hypertension. The Cochrane Library. 2005.
5. Friedman RH, Kazis LE, Jette A, Smith MB, Stollerman J, Torgerson J, et al. A telecommunications system for monitoring and counseling patients with hypertension: impact on medication adherence and blood pressure control. American journal of hypertension. 1996;9(4):285-92.
6. Lim FS, Foo M, Kanagalingam D, Lim R, bte Bahadin J, Tan KL, et al. Enhancing chronic disease management through telecare–the Singapore Health Services experience. Journal of Telemedicine and Telecare. 2007;13(suppl 3):73-6.
7. Wilson PW, D'Agostino RB, Levy D, Belanger AM, Silbershatz H, Kannel WB. Prediction of coronary heart disease using risk factor categories. Circulation. 1998;97(18):1837-47.
8. Ware Jr JE, Snyder MK, Wright WR, Davies AR. Defining and measuring patient satisfaction with medical care. Evaluation and program planning. 1983;6(3):247-63.
9. Green BB, Ralston JD, Fishman PA, Catz SL, Cook A, Carlson J, et al. Electronic Communications and Home Blood Pressure Monitoring (e-BP) study: Design, delivery, and evaluation framework. Contemporary Clinical Trials. 2008;29(3):376-95.
10. Myers MG, Godwin M, Dawes M, Kiss A, Tobe SW, Grant FC, et al. Conventional versus automated measurement of blood pressure in primary care patients with systolic hypertension: randomised parallel design controlled trial. BMJ. 2011;342.
11. VanVoorhis CRW, Morgan BL. Understanding power and rules of thumb for determining sample sizes. Tutorials in Quantitative Methods for Psychology. 2007;3(2):43-50.
